# The Infectious Diseases Society of America Lyme guidelines: a cautionary tale about the development of clinical practice guidelines

**DOI:** 10.1186/1747-5341-5-9

**Published:** 2010-06-09

**Authors:** Lorraine Johnson, Raphael B Stricker

**Affiliations:** 1 California Lyme Disease Association, Ukiah, CA, USA; 2 International Lyme and Associated Diseases Society, Bethesda, MD, USA

## Abstract

Flawed clinical practice guidelines may compromise patient care. Commercial conflicts of interest on panels that write treatment guidelines are particularly problematic, because panelists may have conflicting agendas that influence guideline recommendations. Historically, there has been no legal remedy for conflicts of interest on guidelines panels. However, in May 2008, the Attorney General of Connecticut concluded a ground-breaking antitrust investigation into the development of Lyme disease treatment guidelines by one of the largest medical societies in the United States, the Infectious Diseases Society of America (IDSA). Although the investigation found significant flaws in the IDSA guidelines development process, the subsequent review of the guidelines mandated by the settlement was compromised by a lack of impartiality at various stages of the IDSA review process. This article will examine the interplay between the recent calls for guidelines reform, the ethical canons of medicine, and due process considerations under antitrust laws as they apply to the formulation of the IDSA Lyme disease treatment guidelines. The article will also discuss pitfalls in the implementation of the IDSA antitrust settlement that should be avoided in the future.

## Introduction

'Without health, there is no happiness'. Thomas Jefferson

'The strong do what they can; the weak endure what they must'. Thucydides

With the trend toward centralized medical decision making and evidence-based medicine, clinical practice guidelines are becoming a key factor in the practice of medicine and are increasingly relied upon by physicians seeking treatment guidance. However, when guidelines panels fail to conscientiously safeguard the integrity of the guideline development process, the quality of guidelines may be eroded and fall short of the primary goal of guidelines--namely improving patient outcomes [1]. The Connecticut Attorney General's antitrust investigation into the Lyme disease treatment guidelines development process of the Infectious Diseases Society of America (IDSA) underscores the importance of these problems and the need for guidelines reform [2].

Guidelines have become a way to drive the medical standard of care that governs physician conduct. When inflexible guidelines are adhered to by insurers, government agencies, medical societies and hospitals, guidelines can essentially create a *de facto *regulatory scheme fraught with economic, legal, and patient care consequences. Accordingly, guidelines, particularly those formulated by medical societies that are considered dominant under antitrust laws, hold enormous influence over the practice of medicine, creating a situation that is ripe for abuse [3]. For example, third parties such as the insurance industry and pharmaceutical companies whose commercial interests may be affected by guidelines may seek to influence the guidelines development process through the use of 'key opinion leaders' (typically academic researchers) who serve on guidelines panels. The enormous influence of clinical practice guidelines also creates the potential for the guidelines of a dominant medical society to be used competitively against less influential medical societies.

The primary goal of clinical practice guidelines is to improve patient care. However, in general, patient interests are not directly represented on guidelines panels. Guidelines that limit patient treatment options may essentially set public policy without the benefit of public debate or the participation of significant stakeholders. When divergent treatment approaches exist and guidelines fail to acknowledge these or provide treatment options, they may deprive patients of the right to make the treatment choices that lie at the heart of autonomy.

Despite the potential for clinical practice guidelines to restrict patient care, to promote the interests of commercial third parties, and to be misused by medical societies against their competitors, guidelines have remained largely unregulated. The potential for guidelines abuse has sparked calls for reform in recent articles highlighting issues that may undermine the validity of these guidelines. These issues include conflicts of interest on guideline panels, the relative paucity of evidence supporting many guideline recommendations, the over-reliance on expert opinion, the false appearance of unanimity of opinion presented in guidelines, the failure to acknowledge legitimate controversy, the lack of rigorous external peer review when medical specialty societies self-publish guidelines, and the interference with the legitimate exercise of clinical judgment created by inflexible recommendations [1,4-6].

Traditionally, there has been no legal remedy for a flawed guideline development process. However, in November 2006, the Attorney General of Connecticut launched a ground-breaking antitrust investigation into the development of Lyme disease treatment guidelines by IDSA, one of the largest medical societies in the United States [2]. As discussed below, antitrust law is concerned with abuses of power by those who have it. Ultimately, antitrust laws focus on actions by 'dominant' organizations that constrain consumer choice and employ 'exclusionary conduct' to suppress the views of competitors. In medicine, guidelines that limit the access of patients to treatment options may constrain consumer choice. The emphasis of antitrust law in the context of treatment guidelines is on the fairness or integrity of the guideline development process.

The Connecticut Attorney General's investigation found significant irregularities in the IDSA Lyme guideline development process, including significant conflicts of interest among the guidelines panel members. These issues parallel those underlying recent calls for guideline reform. The settlement of the investigation required IDSA to review its guidelines with a new panel that is free from these conflicts. The findings of that investigation, the settlement of that investigation, and the mechanics of the settlement process were discussed in detail in a previous article by the authors [7]. This article will examine the recent calls for guidelines reform in the context of the IDSA antitrust investigation and settlement and the interplay between medical ethics canons, guideline reform, and due process considerations under antitrust laws.

## The Lyme disease controversy and IDSA guidelines

The diagnosis and treatment of Lyme disease is highly controversial, especially when the initial tickborne illness is not recognized and symptomatic disease persists [8]. The level of disability of patients with persistent Lyme disease symptoms is equal to that of patients with congestive heart failure [8]. The spirochetal bacterium that causes Lyme disease was first identified in 1982, and the state of Lyme disease science is best described as emerging, with many gaps in research remaining to be filled. The handful of treatment studies for Lyme disease have faced significant design challenges, produced conflicting results and involved small sample sizes. Consequently the studies suffer from limited generalizability when applied to a clinical patient population that is heterogeneous [9].

In the past decade, two opposing camps have emerged in the battle over the tick-borne illness. One is represented by IDSA, which maintains that Lyme disease can be treated with short courses of antibiotics and that persistent infection is rare or non-existent [10]. The opposing camp is represented by the International Lyme and Associated Diseases Society (ILADS), which notes the high failure rate of short courses of antibiotics for disseminated Lyme disease and maintains that the underlying infection may persist in a large number of patients and require prolonged antibiotic therapy [11,12]. The controversy between the two camps has been labeled the 'Lyme Wars' [13].

Despite the emerging state of the science and considerable uncertainty about the disease, the playing field for this debate between the warring factions has been far from even. IDSA is widely recognized as the preeminent infectious disease specialty society in the United States and publishes two of the three most influential infectious disease journals [14,15]. Its members exert strong influence on peer review for medical journals by serving as peer reviewers and editors. For instance, one member of the IDSA Lyme guidelines panel provides peer review for over 30 medical journals [16]. IDSA also has considerable power over antibiotic treatment protocols in hospitals, which typically employ an infectious disease consultant to establish and monitor the use of antibiotics in the hospital. Hence, the ability to identify and target noncomplying physicians may 'induce hospitals to deny or revoke hospital privileges for physicians who do not comply with (IDSA) guidelines,' thus permitting IDSA-affiliated physicians to act as gatekeepers for hospital staff privileges [17].

IDSA guidelines may serve as a proxy for the standard of care applied by medical boards in unprofessional conduct actions. Before commencing an action, medical boards commonly refer potential actions out for 'expert' review by members of IDSA [17]. In addition, IDSA-affiliated physicians testify against non-complying physicians in unprofessional conduct actions [18]. When organizations wield this amount of power, they can run afoul of antitrust law if they attempt to exclude competing viewpoints, fail to control conflicts of interest, and restrict consumer choice.

In 2005, IDSA seated a panel to develop revised Lyme treatment guidelines. Under the prior IDSA Lyme guidelines published in 2000, a number of physicians had been subjected to unprofessional conduct actions for failure to comply with the guidelines, insurance companies were denying patient reimbursement for treatment that was not in compliance with the guidelines, and patients were having difficulty finding physicians willing to treat their illness [7]. A number of physicians with divergent viewpoints, including some members of IDSA, applied for a seat on the panel, but they were rejected by IDSA ostensibly on the basis that the panel was full, although the number of panelists was subsequently increased [2].

The revised Lyme guidelines were published in October 2006 [10]. As most patients and their treating physicians feared, the guidelines restricted the management of patients by the treating physician. Nineteen members of the U.S. Congress sent a letter to the Centers for Disease Control and Prevention (CDC) requesting a review of guidelines that 'had the potential to effectively shut down' treatment of chronic Lyme disease [19]. As the authors have noted elsewhere, because the guidelines do not provide for treatment options or the exercise of clinical judgment by physicians, they may be viewed as a mandatory standard of care by medical societies, government agencies, and insurance companies that may adopt the guidelines based on the reputation, specialty dominance, and distribution power of IDSA [7].

IDSA contends that it does not intend for its guidelines to be applied as mandatory treatment protocols [20]. However, the guidelines do not provide for treatment options or the exercise of clinical discretion, and they fail to acknowledge the existence of divergent treatment approaches [10]. Although the guidelines contain the usual formulaic disclaimer regarding clinical judgment, this disclaimer does nothing to overcome the treatment restrictions that are inherent in the guidelines. Other medical societies expressly provide for treatment options when guidelines are not mandatory [21]. Moreover, IDSA's assertion that the guidelines are not mandatory is at odds with two practical realities: IDSA members testify against physicians who fail to comply with the IDSA Lyme guidelines, and IDSA opposes state legislation designed to protect its competitors from unprofessional conduct actions based on those guidelines [14,18].

Matters ultimately came to a head in Connecticut, which has the highest incidence of reported Lyme cases in the nation and is ground zero for the 'Lyme Wars'. The California Lyme Disease Association (CALDA) spearheaded a national effort with other advocacy groups, including the national Lyme Disease Association and Connecticut-based Time for Lyme. Focusing on antitrust law as a vehicle to address the restrictive guidelines, the groups approached the Attorney General of Connecticut, Richard Blumenthal, who responded by launching an antitrust investigation.

The authors of this article stand on the minority side of this debate, and the first author was instrumental in developing the antitrust theory with a colleague, Richard Wolfram, a New York-based antitrust attorney, and in presenting that theory to the Connecticut Attorney General. Each of the authors serves on the board of directors of CALDA and ILADS, although this article has been written in their individual, rather than organizational capacities. In addition both presented testimony before the IDSA Lyme guidelines review panel.

## The application of antitrust law to the development of treatment guidelines

Treatment guidelines are intended to influence physician practices and may be used to discourage competing viewpoints. A recent article in the *Journal of the American Medical Association *by Sniderman and Furberg explains the competitive aspects of guidelines: 'By favoring one test over another, or one therapy over another, guidelines often create commercial winners and losers, who cannot be disinterested in the result and who therefore must be separated from the process' [4]. Antitrust laws are intended to 'promote competition and level the playing field in our marketplace' [22]. IDSA is considered dominant under antitrust laws and its guidelines are considered authoritative in the area of infectious diseases. When Attorney General Blumenthal launched the antitrust investigation against IDSA, he stated that the purpose of the investigation was to determine whether the IDSA guidelines 'constrain choices by patients or doctors in a way that could be anticompetitive'[23]. Although antitrust issues have arisen in other contexts in medicine in the past, the Connecticut investigation marks the first time that the development process for treatment guidelines has come under antitrust scrutiny.

The investigation arose out of the doctrine of 'standard-setting' in antitrust law. Standards are pervasive; they may be simple or complex, and may include safety standards for products. Typically, in the process of setting standards, firms compete in an organized, structured process for their technology or products to be incorporated into a standard. This process preempts market choices, but usually passes antitrust muster because it yields pro-competitive efficiencies that benefit consumers.

According to Richard Wolfram, the antitrust attorney in New York who was involved in the investigation, 'Because standard setting by competitors supplants competition, *the process must be fair, open, and not subject to any bias *by participants with economic interests in stifling completion, especially when the standard-setting is done by an association...that is highly influential or dominant in the relevant market place'[17]. Although IDSA claims that the antitrust investigation is a case of the government meddling with medicine, antitrust law is not concerned with calling the science; instead it requires that the *development process *should be fair, non-exclusionary, and not tainted by conflicts of interest [17].

## Recent calls for guidelines reform

Flaws in the development of guidelines that are related to conflicts of interest, bias among the members of the guidelines panel, and suppression of competing viewpoints were central to the antitrust investigation of IDSA by Attorney General Blumenthal. Moreover, these flaws mirror the growing list of problems with evidence-based guidelines in general, which has led many to call for reform in how guidelines are developed [4]. In their article in the *Journal of the American Medical Association*, Sniderman and Furberg highlighted critical deficiencies in the current process:

• Conflicts of interest of 'experts' on guidelines panels may substantively drive the content of the guidelines in a manner that does not hold the interest of the patient paramount.

• A paucity of high-level evidence and an overreliance on 'expert opinion' may result in the formulation of guidelines that merely replace one 'authority-based' system with another.

• Legitimate controversy may be suppressed by artificially 'unanimous' panel recommendations and by the exclusion of divergent viewpoints from the panel.

• Specialty medical societies, which use guidelines to expand their competitive sphere of influence, may publish guidelines in their own journals essentially 'as-is,' without submitting them to the type of independent, external peer review that might vet issues of bias or conflicts of interest on the part of guidelines panel members [4].

All of these aspects undermine the credibility of guidelines and permit personal bias to determine the care of patients -- the very problem that evidence-based guidelines are intended to avoid. Indeed, these deficiencies may undermine the ethical foundation of medicine, which requires 'physicians to put the needs of patients ahead of personal gain, to deal with patients honestly, competently, and compassionately, and to avoid conflicts of interest that could undermine public trust in the altruism of medicine' [24].

The integrity of medicine, and, ultimately, its value to society depends upon recognizing and safeguarding this ethical foundation. Sniderman and Furberg highlight the most important components of that ethics foundation in the context of guidelines: conflicts of interest, overreliance on expert opinion, and failure to acknowledge legitimate controversy. Distilled down to their essence, these issues are based on two ethical principles: the need to hold the interests of the patient above other commercial interests and the need to preserve the 'treatment choice' that gives life to the ethical principle of autonomy.

The ethical obligation to hold patient's interests paramount can be compromised by conflicts of interest resulting from financial ties of the panel members. The ethical obligation to respect patient autonomy depends upon the preservation of treatment choice for patients and can be jeopardized when guidelines fail to acknowledge legitimate controversy and do not provide treatment options. Choice is also jeopardized when there is a paucity of evidence and evidence gaps are filled by the 'expert opinion' of the panel. The problems that arise from conflicts of interest and failure to preserve treatment choice are furthered when the medical specialty societies that sponsor and publish these guidelines fail to adequately police these risks or have industry ties themselves. The issues regarding guidelines development and conflicts of interest, quality of evidence, and patient choice are discussed in more detail below.

### Conflicts of Interest

One of the paramount deficiencies identified by Sniderman and Furberg in guidelines development arises from conflicts of interest. The Institute of Medicine (IOM) defines a conflict of interest as 'a set of circumstances that creates a risk that professional judgment or actions regarding a primary interest will be unduly influenced by a secondary interest' [25]. This determination requires identifying a primary interest and a secondary interest. The primary goal of medicine that should stand at the center of treatment guidelines is to '[improve] health by providing beneficial care to patients' [25]. Secondary interests 'may include not only financial gain but also the desire for professional advancement [and] recognition for personal achievement' [25]. This emphasis on primary versus secondary interests arises from the divergent goals of medicine (improving patient health outcomes) and commerce (ensuring a financial return to shareholders) [25].

Not all conflicts are of equal severity. The IOM suggests that conflicts should be assessed by considering both the likelihood of adversely affecting the primary interest and the seriousness of the harm caused by the conflict:

Likelihood depends on the value of the secondary interest, the scope of the relationship between the professionals and the commercial interests, and the extent of discretion that the professionals have. Seriousness depends on the value of the primary interest, the scope of the consequences that affect it, and the extent of accountability of the professionals [25].

Because they may affect multiple patient care decisions and criteria for insurance coverage, clinical practice guidelines are considered by the IOM to have a serious potential for harm [25]. This is compounded by the fact that guidelines panels have not usually been held to be legally accountable to patients under malpractice laws because the duty of care that creates liability exposure only arises in the context of a direct physician-to-patient relationship [26]. Discretion of those on guidelines panels increases when the scientific evidence base is weak and the panel elects to plug evidence gaps with 'expert opinion' rather than acknowledging the uncertainty and providing treatment options. These factors increase the risk and seriousness of harm to patients and emphasize the importance of managing conflicts of interest. Parenthetically, the growing importance of guidelines in public policy may increase the application of negligence and strict liability law to their development process [26].

Unfortunately, conflicts of interest on guidelines panels are common. It is not unusual for 'expert' panels to include 'key opinion leaders', usually academic researchers for whom industry ties are vital to ensure the research funding on which their careers depend [27]. Choudhry and colleagues found that 87 percent of participating 'experts' had financial ties to pharmaceutical companies, and 59 percent had ties to companies whose products were considered in the guidelines authored by the 'experts' [27]. The roll call of guidelines formulated by 'conflicted panels' that appear to further the interests of pharmaceutical companies includes those for the treatment of sepsis, anemia among kidney patients, and high cholesterol [28]. A *New York Times *article commented that a conflicted guidelines panel that adopted an industry-friendly new definition of high blood pressure 'illustrate[d] connections -- among the pharmaceutical industry, academic physicians and societies that formulate opinion -- that can ultimately affect patient treatment,'[29] and called this 'the monetarization of medicine' [29].

A central value of research is the pursuit of objective, unbiased information, and many 'key opinion leaders' believe that they can rise above commercial conflicts of interest [27]. Common sense and recent investigations indicate the opposite [30]. When money enters the equation, the nature of the debate simply changes: for example, Choudhry and colleagues found that research articles have either been published or discretely shelved based on ties to industry [27].

Choudhry and colleagues argued that the subtle influence of money on 'key opinion leaders' forms the basis of a substantial portion of pharmaceutical marketing to promote the industry's interests [27]. Nonetheless, they noted that the vast majority of researchers believe that at least their own research is not compromised by monetary ties with industry [27]. The Association of American Medical Colleges reported that, in contrast to deliberate corruption, many ethical problems in the professions are based on unconscious bias that permeates professional conduct; that is, professionals with conflicts of interest may selectively seek out information, may interpret information in a biased fashion, and may easily be derailed from good intentions, but are typically unaware of these effects [31].

When researchers dominate the panels that write guidelines, they may bring their research biases with them. Ernst and Canter describe the adverse effects that bias may have on research:

Bias is ubiquitous, and medical research is no exception. From the very outset, investigator bias can influence the general attitude towards a research project....The biased researcher, however, has preconceived ideas and is likely to approach a project to prove a point. For example, a researcher who is convinced of a particular treatment or, worse, has a vested interest in it, could misuse science to demonstrate the efficacy of his therapy. Equally, an investigator with a preconceived negative attitude towards a particular intervention can set out to disprove its efficacy [32].

While some bias is unavoidable, biases introduced by conflicts of interest frequently are and should be avoided to the extent possible.

Academic researchers who sit on panels may have conflicts of interest not only in the form of industry ties (particularly if they are regarded as 'key opinion leaders'), but also other competing financial interests identified by the IOM, like career advancement or recognition of personal achievements [25]. For example, citing their own research in guidelines may promote both future research funding and further their academic careers [27]. A report from the Institute for Clinical Research and Health Policy Studies at Tufts University describes researchers who have staked their careers on a particular theory and concludes that they 'are likely to resist accepting that the whole field in which they have spent their lives is a null field'[33]. These factors may impair the ability of guidelines panel members to critically select and evaluate research findings in an unbiased fashion.

In addition, serving on treatment guidelines panels is part and parcel of being a 'key opinion leader'. In his pharmaceutical marketing textbook, Ronald Evens, a professor at University of Florida, College of Pharmacy, states:

[I]t is paramount to identify national and regional influencers, that can help guide registrational trial program development, conduct solid and timely clinical research, generate leading disease state publications, *help draft treatment guidelines*, and begin cultivating potential speakers to help leverage clinical trial product use immediately post drug approval [34].

Conflict of interest policies typically screen for conflicts within the last year or two years, and 'key opinion leader' relationships with industry may span a career [35]. For example, one researcher on the IDSA Lyme guidelines panel, Allen Steere, has relationships spanning more than a decade with Lyme vaccine manufacturers [10,36,37]. Although researchers often develop a known expertise and significant influence in a research area, these 'key opinion leaders' may change their affiliations with pharmaceutical companies over a given time frame. Thus it may be unrealistic to assume that conflicts of interest among 'key opinion leaders' are adequately reflected by a time-delimited inquiry.

In addition to the conflicts of interest of individual panel members, the medical society that sponsors and publishes the guidelines may have conflicts of interest [4]. Industry funding of medical societies is extensive [5]. A recent IOM report reviewed the budget of the American Association of Family Physicians for the fiscal year 2006-2007. Less than 38% of its $80 million budget came from membership dues and services, while 42% came from the pharmaceutical industry, (60% from advertising in the academy's journal and 13% from exhibit fees) [25].

Although medical societies profess to operate for the public good, Noble and colleagues observed that '[g]uidelines promulgated by a particular medical society generally reflect the specific concerns of its members' [26]. The behavior of societies may not only further their own economic interests, but may also take advantage of opportunities to 'sit in judgment of their competitors' [38]. Treatment guidelines expand the society's sphere of influence, and, according to the IOM, medical specialty societies regard guidelines as one of the most valued services they provide [25]. These factors suggest that the interests of medical societies (and those of the individuals serving on a guidelines panel) may not align with the best interests of patients, their professional competitors, or the interests of the broader public [5].

Moreover, as Sniderman and Furberg noted, 'Few associations submit the final products of the guideline process for external review before they are accepted and, therefore, in a limited but real sense, the committee, which is a creation of the organization, becomes the final arbiter of its process,' bypassing 'one of the core processes of science,' the type of review that might hold these self-interests in check [4].

How should conflicts of interest issues be addressed in the guideline development process? In testimony before the Institute of Medicine, Merrill Goozner, Director of the Integrity in Science Project at the Center for Science in the Public Interest, emphasized the need to maintain strong boundaries between those who determine how medicine gets practiced and researchers who conduct industry-funded research [39]. The IOM agreed:

Given the important role that clinical practice guidelines play in many aspects of health care, it is important that these guidelines be free of industry influence and be viewed by clinicians, policy makers, patients, and others as objective and trustworthy [25].

But what if conflicts of interest are the price of expertise, as is often argued? The IOM provided further guidance on this point.

If groups conclude that participants with conflicts of interest are essential to provide the necessary expertise, they should demonstrate to the public that they have made a good faith but unsuccessful effort to find individuals with the required expertise and without conflicts of interests. They should preclude individuals with conflicts of interest from chairing guideline development panels, restrict the number of individuals with conflicts of interest on panels to a distinct minority (e.g., to 25-30 percent of the membership), and prohibit members with conflicts of interest from drafting and deciding specific recommendations [25].

Hence, 'key opinion leaders' with industry-related conflicts of interests ideally should be excluded from guidelines panels. Where this is not possible, the influence of these individuals should be regulated by limiting participation to no more than 25-30% of the panel and otherwise restricting the leadership role of those with conflicts of interest.

### Quality of Evidence

The implicit goal of evidence-based medicine is the provision of medical care that is grounded in science. However, the medical research base lags far behind in providing the clinical evidence that physicians need to treat patients, and the majority of recommendations in most treatment guidelines are not based on high-quality medical research studies [40].

Given our reliance on evidence-based guidelines, this shortfall in evidence is disturbing [6]. The IOM reported that '[a] review of guidelines in the National Guidelines Clearinghouse reveals recommendation after recommendation that is supported by weak, mixed, or no evidence' [25]. McAllister and colleagues found that only 68% of the recommendations in guidelines cited randomized controlled trials, and less than 30% were high-quality studies that could be applied to the patient population [40].

At issue is not only the level of the evidence that is cited (for example, controlled studies versus non-controlled studies), but also the internal and external validity of the research, including the size of samples, the endpoints that are evaluated, and the generalizability of the study to the patient population in the medical community at large. Ioannides and colleagues' analysis of research studies concluded that 'for most study designs and settings, it is more likely for a research claim to be false than true' [33].

The problem when the 'evidence' in evidence-based medicine does not deliver is the misperception, as Sniderman and Furberg noted, that 'guidelines are evidence based, not opinion based, and therefore their conclusions flow directly from the conclusions of studies' [4]. Bias created by conflicts of interest on guidelines panels is exacerbated when evidence is weak. Under these circumstances, the real question is not what is 'right', but who decides.

Although evidence based medicine regards the randomized controlled trial as the gold standard, many forms of medical evidence exist. Scott Sehon of the Department of Philosophy at Bowdoin College describes the available broader evidence base:

[C]linical experience, observational studies, and RCTs have much in common. All are attempting to ascertain the safety and efficacy of interventions, and all do so by trying the intervention and noting the results....In each case, we will observe the treatment received by a patient, and then we will observe the outcome or endpoint for each patient [41].

With each of these three forms of evidence, knowledge is based on probability. Hence, to the physician 'knowing a diagnosis means. . .the diagnosis is highly probable [but] not absolute'[42]. The same holds true for observational studies and RCTs, which are based on statistical averages that may or may not apply to a heterogeneous patient population. In any event, regardless of the pretreatment probabilities and the type of evidence employed, the 'absolute' can only be determined by the response of an individual patient to a specific treatment.

The issue of which evidence base should carry more weight in a particular recommendation is important. Put another way, the question is whether centralized medical decision making or individualized care should be given more weight. When the base of evidence is weak, non-existent, or conflicting, the treating physician may become the proverbial one-eyed king in the land of the blind. The clinician alone holds a key to the puzzle that is unavailable to members of a remote guidelines panel: patient histories, physical examination findings, and the patient's responsiveness to treatment. The American Academy of Pediatrics directly tackled this issue in its guidelines on creating guidelines: 'When the evidence is of low quality and the benefit-harm equilibrium is balanced, guideline developers generally should not constrain the clinician's discretion by making a recommendation but instead should designate acceptable alternatives as options'[21].

### Patient Choice

Evidence shortfalls that are filled with 'expert opinion' can take treatment choices out of the hands of treating physicians when clinical discretion is restricted and out of the hands of patients when treatment options are excluded from guidelines. Preservation of treatment options is a necessary underpinning to patient autonomy. Guideline panels that fill in evidentiary gaps with 'expert opinion' should be mindful of not interfering with medical decisions that properly belong to the patient. Drawing the line between paternalism and patient autonomy is a subtlety that pervades medical care.

In a clinical setting, the patient can respond to undesired paternalism by exercising 'voice or exit strategies' [43]. Typically, the physician describes the choices and recommends that the patient follow a particular treatment approach. The patient can discuss concerns (voice) and the physician may then modify the treatment recommendation to reflect the patient's individual values [43]. Alternatively, if the physician does not offer treatment options or if the patient does not improve under his care, the patient can exercise his right to leave the relationship (exit) and seek help elsewhere [43]. Either way, the physician is accountable and bears the consequences of his actions, both legally and economically. In guidelines development, there are no voice or exit approaches available to the patient. Hence, guidelines panels need to be particularly reticent about interfering with treatment choices or clinical discretion.

The boundary between treatment recommendations that should be made by the panel and treatment options that should be preserved for patients depends upon (1) the quality of the evidence and (2) whether treatment decisions involve trade-offs between risks and benefits that depend on the values of the patient [44]. Because of the lack of voice and exit options for patients in guideline treatment recommendations, a high degree of certainty should be required before interfering with patient choice and clinical discretion. In his treatise on participatory democracy and bioethics, Thomas May at the Medical College of Wisconsin's Center for the Study of Bioethics addressed the question of choice in the face of medical uncertainty. He observed that 'there is no question that amputation is the appropriate action in extreme cases of gangrene' and concluded that the 'role of bioethics is precisely that of calling into question whether any treatment is appropriate without question' [45]. In the absence of such certainty, May concluded that it is important to preserve choice and to allow the individual whose life is most affected by that choice, the patient, to exercise autonomy of decision.

This comports with the American Medical Association's code of ethics, which states: 'The principle of patient autonomy requires that competent patients have the opportunity to choose among medically indicated treatments and to refuse any unwanted treatments' [46]. As May explained:

Determining 'appropriate' treatment, then, is itself a determination that includes, among other things, a value judgment. As we have seen, what is right for one patient may not be right for another. Whether a given treatment is appropriate depends on whether the potential benefits of that treatment outweigh the burdens it imposes on the patient. This judgment requires that we consider the patient's perspective in assessing the benefits and burdens of treatment. To fail to do so is inconsistent with a liberal constitutional society and with the rights of a patient in such a society [45].

Ezekiel Emanuel, current advisor to President Obama, explains why choice, autonomy, and informed consent are central issues in medical ethics:

Most health policy analysts...see choice as essential because individuals are the best judges of their own interests. While individuals may not always be correct, they have more intimate knowledge of themselves, more reason to get it right, and more motivation to be dogged in pursuit of their interests. When choice is restricted or relegated to someone else, there is a high chance that individuals will be prevented from realizing their interests or their interest will be sacrificed to someone else's interest. This view is commonly cited by courts and others as one of the justifications for informed consent [43].

Paternalism is generally rooted in the belief that one is 'right' and that intervention is necessary to protect the patient from making a 'bad' decision. But, as David Buchanan at the Amherst School of Public Health and Health Sciences, explains, good intentions do not justify interference with personal liberty:

Dworkin defined paternalism as 'interference with a person's liberty of action justified by reason referring exclusively to the welfare. . .of the person being coerced'. Paternalism is the usurpation of decision-making power, by preventing people from doing what they have decided, interfering in how they arrive at their decisions or attempting to substitute one's judgment for theirs. . .The moral concern is that the presumption that one is right, and therefore justified in seeking to override other people's judgment, constitutes treating them as less than moral equals [47].

With autonomy '[t]he critical point is being in the position of deciding, not being decided for' [47]. As May pointed out, the fact that an expert may disagree with the patient is beside the point; autonomy only becomes a real issue when an expert disagrees with the patient [45]. Hence, one of the essential tasks of a guidelines panel is to identify and preserve 'choice' for the patient. Choice can be preserved by acknowledging divergent viewpoints on treatment, providing treatment options, or deferring to clinical judgment. Guidelines of highly influential medical societies need to be crafted with care not to interfere with patient choice.

When evidence is clear, well developed and uncontested, the need to preserve choice is often not an issue. However, it is essential that treatment choices be preserved when evidence is weak, mixed or not yet developed. As was noted in the discussion earlier, the lack of a strong evidentiary base supports the decisive role of clinical judgment exercised by the clinician rather than the remote recommendations of a guidelines panel.

Beyond this, however, what should be done to preserve legitimate treatment choice for the patient? Choice arises out of a diversity of viewpoints. As Sniderman and Furberg pointed out, '[b]ecause gaps in the evidence are inevitable, they must be filled in with judgments, and judgments tend to preserve previous positions. Thus, what is to be decided is often already decided with the selection of the deciders' [4]. Hence, guidelines panel constitution may well determine the range of treatment options available to patients.

Hotly contested areas of medicine such as the 'Lyme Wars' arise most often, as Atkins and colleagues noted in *Health Affairs*, 'when evidence is weaker, outcomes are less certain, and parties disagree about the risks of acting in the face of uncertainty' [48]. The IOM addressed the issue of guidelines panel constitution and concluded:

The inclusion of individuals with a range of relevant professional and other backgrounds on guideline development panels can help check financial, professional, and other sources of bias. . .[and] promote the fuller consideration of potential outcomes, relevant evidence, and aspects of implementation [25].

The concept of including a diverse panel is to ensure diverse viewpoints--that is, to ensure that the interests of different stakeholders are represented to the extent that their interests are legitimate and central to the practice of medicine. According to the IOM, 'clinical practice guidelines lie at the intersection of medical research, education, and practice' [25]. It follows that researchers and community physicians should be included on the panel. Other stakeholders include patients, insurance providers and pharmaceutical companies.

Most evidence-based guidelines panels are filled with academic researchers rather than with community physicians. In part, this represents a hegemonic shift away from commercially independent physicians who have traditionally controlled medicine, toward academic researchers whose commercial funding as 'key opinion leaders' may frame their viewpoints [49]. As Sniderman and Furberg point out, however, this hegemonic shift may merely transfer power from one authority based expert, the physician, to another authority based expert, the researcher [4]. Clearly researchers offer something of value when they sit on guidelines panels, but their perspective is very different from that of the general practitioner who on a daily basis sees patients who do not conform to research populations and has first-hand experience with the heterogeneity of treatment response, the need to preserve clinical judgment in guidelines, and the importance of providing patients with treatment options. Moreover, as was recognized during the AIDS activist movement, there may be a 'clash between the canons of research [which emphasize protection of research subjects] and the canons of care [which emphasize availability of treatment options]' [50].

As was noted earlier, the standard of care for physicians is driven more and more by treatment guidelines. Before the trend toward centralized medical decision-making and evidence based guidelines, the standard of care was largely determined by the 'consensus' of practicing physicians. This consensus developed slowly over time and reflected the practices of many physicians. Today recommendations of a guidelines panel may serve as a proxy for the consensus of treating physicians in determining and driving the medical standard of care. In fact, '[f]or many clinicians guidelines have become the final arbiters of care' [4]. Thus, it is critical that guidelines not convey a false sense of unanimity when none exists.

Guidelines need to elucidate rather than obscure serious debate regarding diagnosis and treatment since 'the very nature of scientific debate is that no *right *answer has emerged' [4,51]. The only way to assure this transparency is through panel diversity reflecting the range of treatment variation and practice. This means that diverse viewpoints should be represented on guidelines panels, that true voting of recommendations should occur to determine the degree of consensus, and that legitimate controversy should be acknowledged and reflected in the guidelines text.

The other viewpoint that must be reflected in treatment guidelines is that of the patient. The patient's viewpoint is essential to preserve the 'choice' among medical treatment options upon which the right to autonomy rests. When legitimate treatment options are not reflected in guidelines, autonomy has no meaning. Patients may be represented by direct participation on panels or by their treating physicians, and the value of direct participation may vary depending on whether the patient has become an 'expert' on the disease (as those with chronic illnesses sometimes are) [52].

Direct participation on guidelines panels by 'expert' patients is not common, but is growing. For example, the Cochrane Consumer Network currently provides for patient involvement [53]. The role of the consumer is to 'apply ethical principles such as human rights and civil rights', 'comment on choice', identify 'potential conflicts of interest' and to be 'vigilant to many possible sources of bias' [53]. At the end of the day, the goal of patient representation is to preserve the 'choice' and treatment flexibility that makes the exercise of autonomy meaningful. Hence guidelines that recognize clinical judgment, provide treatment options, and safeguard the patient's right to make treatment decisions can achieve this goal without direct patient participation. However, the best way to ensure that these issues are appropriately addressed is to provide a seat at the table to those whose interests are at stake: the patients.

## A case study for abuse of the guidelines development process

The process that IDSA used to develop Lyme treatment guidelines exemplifies many of the issues found unacceptable by commentators calling for reform of the guidelines development process, including uncontrolled conflicts of interest by panel members, a paucity of evidence, overreliance on the panel's 'expert opinion', the exclusion of competing viewpoints, the failure to acknowledge legitimate controversy, and the failure to subject guidelines to broad external review or comment [2]. The links between key issues in the broader debate regarding guidelines and specific problems with the IDSA Lyme guidelines are listed in Table 1. 

**Table 1:** Problems with IDSA Lyme Disease Guidelines

Weakness or Flaw	IDSA Guidelines
Conflict of interest^1^	Key panel members had financial interests related to Lyme disease patents, diagnostic tests, pharmaceutical (vaccines) interests, and insurance consulting fees.^2 ^Citation by panel members of their own research was high (40%).
Overreliance on expert opinion^1^	38 of the 71 recommendations in the guidelines depend on the weakest Level III evidence, namely 'expert opinion'.^3^
Artificial unanimity of recommendations^1^	The panel excluded competing viewpoints voiced by community physicians and members of its rival, ILADS.^2^
Specialty society self-publication^1^	The guidelines were published in an IDSA journal and were not submitted to normal peer review that would include divergent viewpoints. Letters to the editor critical of the guidelines were not published.
Failure to acknowledge legitimate controversy^1^	The controversy over Lyme disease was well known, but physicians with divergent viewpoints were excluded from participation on the panel and the guidelines failed to mention that other treatment approaches exist.^2^
Limitations on the exercise of clinical judgment and failure to provide treatment options^4^	The guidelines impose severe restrictions on the exercise of clinical judgment and fail to provide treatment options despite a weak evidence base.
Academic researchers setting medical protocols^5^	The IDSA panel consisted almost exclusively of academic researchers.^6^

NOTES

1. Sniderman AD, Furberg CD, Why Guideline-Making Requires Reform. *JAMA *2009;301: 429-31.

2. Connecticut Attorney General's Office, 'Attorney General's Investigation Reveals Flawed Lyme Disease Guideline Process, IDSA Agrees to Reassess Guidelines, Install Independent Arbiter,' press release, 1 May 2008, http://www.ct.gov/AG/cwp/view.asp?a=2795&q=414284. (Accessed June 1, 2010)

3. Stricker RB, Johnson L, The Infectious Diseases Society of America Lyme Guidelines: Poster Child for Guidelines Reform. *South Med J *2009;102: 565-6; Keller DM, 'Infectious Disease Treatment Guidelines Weakened By Paucity of Scientific Evidence,' Medscape Medical News, 13 November 2009, http://www.medscape.com/viewarticle/712341 (Accessed June 1, 2010)

4. American Association of Pediatrics (Steering Committee on Quality Improvement and Management), 'Classifying Recommendations for Clinical Practice Guidelines,' *Pediatrics *2004;114: 874-7.

5. Goozner M, 'IOM Urged to Recommend Conflict-Free Zone for Medicine,' http://www.cspinet.org/integrity/watch/200803101.html#3 (Accessed June 1, 2010)

6. Wormser GP, Dattwyler RJ, Shapiro ED, Halperin AJ, Steere AC, Klempner MS, Krause PJ, Bakken JS, Strle F, Stanek G, Bockenstedt L, Fish D, Dumler JS, Nadelman, RB: The Clinical Assessment, Treatment, and Prevention of Lyme Disease, Human Granulocytic Anaplasmosis, and Babesiosis: Clinical Practice Guidelines by the Infectious Diseases Society of America. *Clin Infect Dis *2006;43: 1089-1134.

Many of these issues were identified by Attorney General Blumenthal at the conclusion of his investigation, when he found that:

• IDSA failed to conduct conflicts of interest reviews of panel members with financial interests 'in drug companies, Lyme disease diagnostic tests, patents and consulting arrangements with insurance companies'.

• Gary Wormser, MD, the chair of the panel, was selected inappropriately, 'held a bias regarding the existence of chronic Lyme,' and 'handpick[ed] a like-minded panel without scrutiny by or formal approval of the IDSA oversight committee'.

• The IDSA Lyme disease panel refused to accept or meaningfully consider information regarding the existence of chronic Lyme disease.

• IDSA blocked the appointment of scientists and physicians who had divergent views on chronic Lyme disease who sought to serve on the 2006 guidelines panel by informing them that the panel was fully staffed, even though the membership of the panel was later expanded [2].

The conflicts of interest of the IDSA guidelines panel members were significant and represent the type of ties to industry and patents that are common to key academic researchers in the field. According to the Connecticut Attorney General:

The IDSA's 2006 Lyme disease guideline panel undercut its credibility by allowing individuals with financial interests - in drug companies, Lyme disease diagnostic tests, patents and consulting arrangements with insurance companies - to exclude divergent medical evidence and opinion. In today's healthcare system, clinical practice guidelines have tremendous influence on the marketing of medical services and products, insurance reimbursements and treatment decisions. As a result, medical societies that publish such guidelines have a legal and moral duty to use exacting safeguards and scientific standards [2].

Wormser, who chaired the Lyme guidelines panel, disputed the notion that financial interests could have played a role in the development process: 'There's no potential financial gain for generic drugs that are recommended for short courses. It's inconceivable that anyone would think so. To me it seems disingenuous to make these allegations when they are so absurd' [54]. In Wormser's view, because the panel members were not promoting *medications for treatment*, there could be no conflict of interest.

While conflict of interest abuses related to medications used to treat diseases are well known, conflicts may extend to any commercial area of medicine. As pointed out in a previous forum [7], the IDSA Lyme guidelines restrict the definition of the disease and mandate laboratory testing. Guidelines that restrict the definition of a disease can favor vaccine developers when they serve to increase the reported rate of effectiveness for a vaccine. Guidelines that mandate testing for diagnosis of a disease promote the interests of those who hold patents on diagnostic tests. Guidelines that deny treatment to patients further the financial interests of insurers and their consultants. Attorney General Blumenthal found that the IDSA Lyme guidelines panel had financial conflicts of interest in each of these areas [2].

IDSA recommendations were presented as the unanimous consensus of those on the panel, yet the consensus was achieved by excluding competing points of view and giving voice to a panel that consisted almost exclusively of academic researchers. That academic researchers on the panel were empowered to dictate the practices of not only IDSA members, but also the practices of family physicians, general practitioners, and even competitors throughout the nation -- physicians who were excluded from the panel -- is one example of a hegemonic shift away from clinicians to researchers. Given the exclusion of community physicians from the panel, it is not surprising that the IDSA guidelines do not reflect the value of preserving and deferring to the clinical judgment of the treating physician in the face of scientific uncertainty.

The control of medical guidelines by academic researchers is a problem not only because of the potential for conflicts of interest, but also because one stakeholder should not control medical guidelines that affect many stakeholders. When scientific evidence is lacking or contested, guidelines panels of dominant medical societies should either include representation for all affected stakeholders or should not constrain the rights of stakeholders who are not on the panel by limiting treatment options available to patients or creating legal consequences for competitor physicians.

The IDSA guidelines failed to mention that there were opposing points of view, let alone to reconcile conflicts between its guidelines and the ILADS guidelines, as required by the IDSA protocols for guideline development [55]. Hence, unknowing physicians could read the IDSA guidelines and be unaware that a controversy exists. Moreover, the quality of evidence supporting the guidelines was quite low, due in large measure to the state of the emerging science; the few controlled treatment trials that exist in Lyme disease have yielded conflicting results and suffer from small sample sizes, poor research design, and a lack of generalizability to the broader Lyme patient population [9]. More than half of the recommendations (38 of 71) in the IDSA Lyme guidelines are based on Level III evidence--namely 'expert opinion'. (Table 1). In the context of this poor evidence base, Wormser's 'handpick[ed] like-minded panel' filtered the evidence for the guidelines, interpreted that evidence, and filled evidentiary gaps with the 'expert opinion' of a panel that excluded divergent viewpoints.

Unfortunately, the paucity of evidence noted in the IDSA Lyme guidelines is not uncommon. At the IDSA Annual Meeting in 2009, two independent presentations described the low level of evidence used to support recommendations in IDSA guidelines *across the board*. Lee and colleagues reviewed 30 guidelines published by IDSA between 1994 and 2009, and found that 'more than half were based on Level III evidence, which is from expert opinion or not supported by properly controlled trials'. Furthermore, the study found that only 14% of 'strong' recommendations were based on appropriate Level I evidence. These findings were corroborated by an independent study presented by Khan and colleagues. The second analysis noted that 55% of recommendations from 44 IDSA guidelines were supported only by 'expert opinion' [56].

Notwithstanding these shortcomings, the dominance of IDSA in the marketplace resulted in Lyme disease guidelines that could be treated as mandatory by insurers and the legal system and foreclose treatment options for healthcare consumers. As legal scholars have observed, guidelines may either be used as a shield against liability by those who comply with their protocols, or as a sword against those who do not comply [57]. In the case of Lyme disease, IDSA guidelines have been used as a sword against physicians who do not conform to the IDSA protocols, notwithstanding the fact that these physicians were excluded from the guidelines process[17]. This contentious environment may have a chilling effect on the willingness of physicians to treat patients with Lyme disease. In addition, insurers have relied on the guidelines to deny coverage for non-compliant treatment approaches and to force compliance from 'network' physicians[17].

When guidelines panels substitute their 'expert opinion' for that of treating physicians and reduce treatment options, the patient's right to autonomy -- to be able to choose among treatment options -- is foreclosed, and physicians who practice medicine using their best clinical judgment may risk losing their medical licenses in misconduct actions. The IDSA guidelines development process created a situation rife with antitrust implications.

Even in the throes of the investigation, IDSA resorted to questionable tactics involving 'copycat' Lyme guidelines published by the American Academy of Neurology (AAN) and an article denying the existence of 'chronic Lyme disease' that was published in the *New England Journal of Medicine *[58,59]. Although these publications appeared to provide 'independent corroboration' of the restrictive IDSA viewpoint, closer scrutiny revealed that the AAN and IDSA Lyme guidelines panels featured overlapping members, including the chairmen of both panels, and 11 members of the IDSA guidelines panel were authors of the article published in the *New England Journal of Medicine*. There was no disclosure of the inherent conflict of interest of using overlapping authorship in independent publications to vindicate a guidelines panel that was under investigation. Attorney General Blumenthal found that IDSA portrayed the AAN and IDSA Lyme guidelines panels as 'independent,' even though 'the two panels shared several authors, including the chairmen of both groups, and were working on guidelines at the same time' -- practices that violated the IDSA's own policy on conflicts of interest [2].

## The settlement

After 18 months of investigation, IDSA entered into a settlement with the Connecticut Attorney General's office, under which it agreed to reconstitute a guidelines panel that would be free of conflicts of interest, consider all submitted scientific evidence, and hold a public hearing aired live over the internet [2]. The newly constituted panel would make a determination whether each contested recommendation 'is medically sound in light of all of the evidence and information provided'. A determination to uphold a contested recommendation would require a 75 percent supermajority vote of the panel members. The details of the settlement and the Attorney General's announcement are included as Additional Files 1 and 2 to this article.

Although these measures represented an improvement over the IDSA development process for the 2006 Lyme guidelines, they failed to accomplish the goal of a fair process, as outlined below. A number of comments by two former presidents of IDSA, Donald Poretz and Anne Gershon, implied that IDSA viewed its obligations under the settlement agreement with Attorney General Blumenthal as being a process of merely 'going through the motions,' and that the guidelines would remain unchanged [60-62].

## Continuing concerns

Sniderman and Furberg argue that the developers of treatment guidelines can become 'promoters and defenders' of the guidelines produced under their auspices [4]. When an organization's guidelines are legally challenged -- as they were here -- the validation of the guidelines may become an overarching focus, and to do so quickly and quietly to minimize damage in the press may become a primary goal. Validation of the IDSA Lyme guidelines would permit IDSA to continue to use them to limit competing views of the disease, maintain IDSA's sphere of influence, continue its domination in the marketplace, and reduce potential litigation risk based on the guidelines. In short, the Lyme guidelines review process constituted a classic conflict of interest: IDSA's organizational interests trumped its interest in maintaining the integrity of the review process necessary to provide guidelines that hold patient interests paramount.

The settlement outlined a process that was primarily run by IDSA, which controlled many of the procedural issues. Given what was at stake, would IDSA be able to set aside its self-interest and decide the issues impartially?

As required under the settlement agreement, IDSA held a public application process for the review panel. Early on, patients questioned the appointment of two panel members who had served on previous Lyme guidelines panels in derogation of the settlement agreement requirements [63]. One panel member was removed after intervention by the Attorney General, but the other member remained on the panel [63]. The fact that IDSA could have easily screened panelists for this requirement and failed to do so with two panelists who had written 'IDSA friendly' guidelines in the past raised concerns about IDSA's motivation to conduct a fair review process.

Potential panelists were screened for conflicts of interest by an outside ethicist, Howard A. Brody, Director of the Institute for Medical Humanities at the University of Texas Medical Branch in Galveston. The fact that an ethicist screened panel members for potential conflicts of interest does not mean that these conflicts were removed from the process. For one thing, Brody, who was paid by the IDSA for his services, surprisingly excluded from the panel any physician who earned more than $10,000 per year from treating Lyme disease [63]. The primary interest of patient guidelines is to provide improved patient treatment outcomes, and it is difficult to see how physicians who treat patients on a fee-for-service basis would have a secondary competing interest. Moreover, because most physicians who treat Lyme disease specialize in its treatment and earn more than $10,000 per year doing so, application of this standard alone eliminated the divergent viewpoints of IDSA competitors, allowing IDSA to continue 'sitting in judgment of its competitors', and making it less likely that legitimate controversy would be acknowledged or that patient choice among treatment options would be preserved.

Since medical specialty societies represent their own membership base, should they not be allowed to exclude from the panel the viewpoints of patients and community physicians who hold divergent views? Whether this is legally permissible under antitrust law may depend in part on whether the medical society has sufficient influence and power to constrain consumer choice and harm competitors. Even assuming a medical society wields enormous influence, it can elect to acknowledge legitimate controversy, provide for treatment options, and defer to the clinical judgment of the treating physician when the evidence base is weak.

This is an area where the primary interest of patient care may be at odds with the secondary interest of specialty medical societies in protecting territorial turf and hegemonic dominance over non-specialists. In short, this may represent a conflict of interest for the medical specialty society. The IOM suggested that institutions have separate committees with members from outside the organization to recognize and manage institutional conflicts of interests [25].

The IDSA review panel consisted largely of IDSA members who could give precedence to the reputational interests of their medical society or take on the mantle of being defenders of their society's guidelines. Using the IOM's primary/secondary analysis of conflicts, this constituted a classic conflict of interest. The review panel in turn determined who the presenters at the hearing would be and excluded a physician who authored more than 40% of the ILADS evidentiary submission from presenting at the hearing. The panel also limited the duration of the hearing to one day rather than using the traditional two day hearing process for guidelines [64]. The short hearing was particularly disturbing given the complexity of the issues presented.

On July 30, 2009, the panel heard presentations from those opposing and those supporting the IDSA guidelines. Evidence submitted to the panel by ILADS included more than 300 pages of analysis and roughly 1,300 peer-reviewed research studies opposing the recommendations in the IDSA guidelines [65]. The panel initially said it would have a decision by the end of 2009, but that deadline was delayed.

On February 1, 2010, the Connecticut Attorney General, who was monitoring the panel review process, sent a letter to IDSA that expressed concern over an improper voting procedure adopted by the panel [66]. The procedure used by the panelists essentially flipped a supermajority voting requirement to make revision of the guidelines less likely. For example, according to the letter by the Attorney General, the panel's vote on whether laboratory tests, which ILADS contends are flawed, should be required for a diagnosis of Lyme disease was deadlocked at 4 to 4, indicating that there was no consensus even on a panel that excluded divergent viewpoints [67]. However, the IDSA panel employed different voting requirements and initially concluded that this vote meant that the guidelines did not require revision. According to the Attorney General, the IDSA panel not only violated the voting procedure stipulated in the settlement agreement, it also failed to comply with an internal IDSA memo directing the panel on the proper procedure for voting [67]. A copy of the letter from the Attorney General to IDSA and a copy of the internal memo from IDSA to the panel are included as Additional Files 3 and 4 to this article.

On April 22, 2010, the IDSA review panel released its report [68]. Despite the voluminous testimony presented by ILADS, the panel voted almost unanimously to uphold the guidelines without exception. Carol Baker, the panel chair and former president of IDSA, stated that for 69 guideline recommendations the panel found that each was "medically and scientifically justified in light of all the evidence and information and required no revision." The panel report expressed concern that prolonged use of antibiotics puts patients in danger of serious infection while not improving their condition. The report stated: "In the case of Lyme disease, there has yet to be a single high-quality clinical study that demonstrates benefit to prolonging antibiotic therapy beyond one month." As to the existence of a chronic persistent form of Lyme disease, the panel concluded that "symptoms that are commonly attributed to chronic or persistent Lyme, such as arthralgias, fatigue and cognitive dysfunction, are seen in many other clinical conditions and are, in fact, common in the general population. It would thus be clinically imprudent to make the diagnosis of Lyme disease using these nonspecific findings alone [68]".

To deal with the testing issue where the panel vote was split, the final report concluded that testing was not a true recommendation and therefore did not fall within the parameters of the settlement agreement [68]. The guideline language at issue states: "Diagnostic testing performed in laboratories with excellent quality-control procedures is required for confirmation of extracutaneous Lyme disease." Hence, although the panel originally voted on the testing recommendation with a split vote, when pressed to redo the vote in conformity with the agreement, the panel recharacterized the testing requirement as a "statement" that did not constitute a recommendation. This seemingly transparent attempt to manipulate the vote to ensure a desired outcome, coupled with the exclusion of divergent viewpoints and other process irregularities, show how easy it is for political considerations, such as avoiding legal liability or protecting organizational reputation, to trump impartial scientific review. The Attorney General's office has announced that it will "carefully and comprehensively assess the final report and the review process leading to that report to determine whether the IDSA fulfilled the requirements of our settlement [69]".

## Future considerations

As guidelines play a more central role in managed health care, the issue of fundamental fairness in the development process will be pivotally important, and additional challenges to medical guidelines will arise, particularly where conflicts of interest exist. The integrity of the review process may depend upon whether it is realistic to expect institutional conflicts of interest and impartiality of the process to be effectively managed within the organization whose guidelines are challenged, or whether greater safeguards are necessary. The IDSA review panel process may provide those who subsequently embark on a guideline review process with valuable lessons on process integrity issues that should be addressed.

Alternate conflict resolution approaches might have been more fruitful. Independent oversight through a private conflict resolution advisor may have resolved many of the issues that have arisen. One alternative approach would have been a review conducted by a government agency such as the National Institutes of Health (NIH), which sponsors consensus conferences. However, the NIH has been criticized for its management of both intramural and extramural conflicts of interest in the past [70-72]. Moreover, the relationship between the NIH and IDSA has been close, and IDSA panel members have received a number of NIH grants. In addition, IDSA opposed the adoption of conflict of interest rules for the NIH [72]. Hence, the NIH's ability to act as a neutral arbiter for these types of issues is unclear.

In medicine, professional societies have generally been free to pursue the agendas of their members without considering the impact on competitors or patients. With the increasing importance of guidelines in the environment of universal health care, it can be expected that antitrust laws will become an important factor in guideline development. Antitrust implications may not be relevant when the issues at hand are not controversial or the society considering the guidelines is not considered dominant under antitrust laws. However, when dominant medical societies develop guidelines that are adopted as the standard of care and are used to foreclose treatment options for patients, a different approach is necessary to skirt antitrust scrutiny. Perhaps the best lessons can be learned from the practices of trade associations, typically comprised of competing members of an industry, that have developed considerable experience complying with the due process requirements of antitrust laws.

One of the foremost standard setting organizations is the American National Standards Institute (ANSI). ANSI employs a standard-setting process that provides due process, which it defines as follows:

Due process means that any person (organization, company, government agency, individual, etc) with a direct and material interest has a right to participate by a) expressing a position and its basis, b) having that position considered, and c) having the right to appeal. Due process allows for equity and fair play [73].

ANSI due process guidelines provide that the minimum acceptable due process requirements for the development of consensus include openness, lack of dominance, balance, consideration of view and objections, and a supermajority consensus voting requirement [73]. Openness requires that participation must be open to all affected persons and that it may not be conditional on membership in an organization. Lack of dominance requires that the process not be dominated by any single interest category or organization. ANSI defines dominance as 'a position or exercise of dominant authority, leadership, or influence by reason of superior leverage, strength, or representation to the exclusion of fair and equitable consideration of other viewpoints' [71]. Balance requires a balance of interest from diverse interest categories and does not permit any single interest category to constitute more than one third of the membership of the consensus body. Interest categories include producers, providers and consumers. ANSI protocols require that standard setting organizations record and consider all negative votes accompanied by comments related to the proposal under consideration. Finally, people who are adversely affected by standards developed by ANSI are entitled to appeal.

How would a medical guideline development process developed under ANSI guidelines look? First, the panel would include researchers, community physicians and patients, and none of the participants would need to be members of the medical society. Second, the members of the medical society would not be permitted to dominate the process and could constitute no more than 33% of the panel. Similarly, academic researchers could occupy no more than 33% of the panel. Third, divergent opinion, in the form of negative votes with comments would be recorded and considered. Fourth, votes would require a two-thirds supermajority and decisions would be subject to appeal. Had these requirements been in place when the IDSA panel developed its Lyme guidelines, antitrust issues would not have arisen. Further, had ANSI guidelines been used as a model for the review process, the panel would have been balanced with physicians from ILADS and IDSA, researchers, clinicians and patients.

## Conclusions

Attorney General Blumenthal's investigation 'exposed a deeply flawed process rife with conflicts of interest that improperly excluded alternative views and information' [63]. The application of antitrust law was based on IDSA's dominant position in the marketplace and the foreclosure of treatment options for healthcare consumers. IDSA's response to the guidelines investigation reaffirmed the need for Attorney General Blumenthal to apply antitrust law to insure procedural fairness.

The recent calls for guidelines reform dovetailed with the issues giving rise to the IDSA antitrust investigation. Medical societies have an obligation to acknowledge legitimate controversy in treatment approaches, particularly when the controversy is fueled by a paucity of high-quality evidence. At a minimum, the guidelines issued by a dominant medical society should conform to fundamental rules of due process, fairness, and accuracy. It is critical that the interests of all stakeholders be given a voice, that legitimate controversies be acknowledged, and that treatment options be preserved. The application of antitrust law may provide a much-needed vehicle of reform to prevent future abuses.

As we have argued elsewhere, the exclusion of competing evidence in treatment guidelines is clinically and ethically unacceptable [74,75]. Failure to disclose treatment options violates the principles of patient autonomy and informed consent, which require that treatment options should be disclosed to patients and that treatment decisions should be made with the patient's informed consent [76]. Treatment guidelines should not inhibit patient access to treatment options; rather, guidelines should describe treatment options and default to the clinical judgment of treating physicians in order to maximize the ability of patients to get well.

## Competing interests

The authors serve on the board of directors of CALDA and ILADS, although this article has been written in their individual, rather than organizational capacities. In addition, both authors presented testimony before the IDSA Lyme guidelines review panel.

## Authors' contributions

LJ participated in the conceptualization of the study, performed the research, wrote the initial draft and participated in subsequent rewrites of the manuscript. RBS participated in the conceptualization of the study and participated in subsequent rewrites of the manuscript. Both authors have read and approved the final manuscript.

## Supplementary Material

Additional file 1 **IDSA Settlement Agreement with CT AG**. Settlement Agreement dated April 30, 2008 between IDSA and Connecticut Attorney General. Click here for file

Additional file 2 **CT AG Press Release re: IDSA Settlement Agreement**. Press Release of Connecticut Attorney General dated May 1, 2008 regarding settlement with the IDSA of antitrust investigation and findings of investigation. Click here for file

Additional file 3 **IDSA internal memo re: voting and action plan**. Internal memo dated July 9, 2009 from Jennifer Padberg to IDSA Lyme guidelines review panel regarding voting procedure requirements. Click here for file

Additional file 4 **Letter from CT AG re: panel voting**. Letter from Connecticut Attorney General dated February 1, 2010 to Richard Whitley, President of IDSA, regarding voting irregularities on IDSA Lyme guidelines review panel. Click here for file
