# Supplementary material for: The Infectious Diseases Society of America Lyme guidelines: a cautionary tale about the development of clinical practice guidelines
Source: Philos Ethics Humanit Med. 2010 Jun 9;5:9. doi: 10.1186/1747-5341-5-9 (PMC2901226; doi:10.1186/1747-5341-5-9)
Supplement: Additional file 1 — IDSA Settlement Agreement with CT AG. Settlement Agreement dated April 30, 2008 between IDSA and Connecticut Attorney General. [file 1747-5341-5-9-S1.PDF]

**An Agreement Between the Attorney General of the State of Connecticut and the Infectious Diseases Society of America**

**WHEREAS,** the Attorney General of the State of Connecticut (the "Attorney General") has conducted an antitrust investigation of the Infectious Diseases Society of America ("IDSA"), a nonprofit corporation with tax-exempt status under the Internal Revenue Code, relating to its development and promulgation of clinical practice guidelines for the treatment of Lyme disease (the "Attorney General's Investigation");

**WHEREAS,** the IDSA, represents over 8,000 physicians, scientists, and other health care professionals who specialize in infectious diseases;

**WHEREAS,** the Attorney General contends that his Investigation has uncovered certain significant procedural deficiencies related to the IDSA's development of its 2006 Guidelines for the Assessment, Treatment, and Prevention of Lyme Disease (hereinafter "the 2006 Lyme disease Guidelines");

**WHEREAS,** the IDSA contends that it developed the 2006 Lyme disease Guidelines based on a proper review of the medical/scientific studies and evidence by a panel of experts in the prevention, diagnosis, and treatment of Lyme disease;

**WHEREAS,** the IDSA has cooperated and continues to cooperate fully with the Attorney General's Investigation;

**WHEREAS,** under this Agreement (the "Agreement"), the IDSA will implement an Action Plan under which, *inter alia,* the IDSA will convene a review panel whose task shall be to determine whether or not the 2006 Lyme disease Guidelines should be revised or updated;

**WHEREAS,** the Attorney General and IDSA enter into this Agreement solely for the purpose of resolving all issues related to the Attorney General's Investigation and not for any other purpose, and this Agreement is not intended to be used for any other purpose;

**WHEREAS**, the Attorney General finds the relief and agreements contained in this Agreement appropriate and in the public interest;

**WHEREAS**, without admitting any of the foregoing, the IDSA enters into this Agreement without any court entering any findings of fact or conclusions of law relating to the Attorney General's Investigation;

**WHEREAS**, neither this Agreement, nor any acts performed nor documents executed in furtherance of this Agreement are an admission of liability, or may be used as an admission of liability or of any wrongdoing.

**NOW THEREFORE**, the Attorney General and the IDSA do hereby enter into this Agreement and agree as follows:

1. The IDSA shall adopt an Action Plan in the form attached hereto as **Exhibit 1**. Time is of the essence with respect to all provisions of the Action Plan that specify a time for performance; provided, however, that the foregoing shall not be construed to limit or deprive the IDSA of the benefits of any grace or use period allowed therein.

2. Nothing in this Agreement shall be construed as relieving the IDSA of its obligation to comply with all state and federal laws, regulations, or rules, nor shall any of the provisions of this Agreement be deemed permission to engage in any act or practice prohibited by such laws, regulations, or rules.

3. By entering into this Agreement, the Attorney General resolves all investigations, issues, claims, and causes of action against the IDSA, its officers, employees, directors, members, and nonmember panel members, consultants, and reviewers who served on its Lyme disease guidelines committees relating to the Attorney General's Investigation and releases and discharges the IDSA, its officers, employees, directors, members, and nonmember panel members, consultants, and reviewers who served on its Lyme disease guidelines committees

from any and all claims or causes of action – whether known or unknown – arising prior to the date of this Agreement relating to the Attorney General's Investigation, the 2006 Lyme disease Guidelines, or the 2000 Lyme disease Guidelines. Notwithstanding this paragraph or any other provision of this Agreement, this Agreement shall in no way be interpreted to release any person with respect to claims that may arise after the date of this Agreement.

4. The Attorney General may make such application as appropriate to enforce or interpret the provisions of this Agreement (including the Action Plan) or, in the alternative, maintain any action within his legal authority for such other and further relief as the Attorney General may determine in his sole discretion is proper and necessary for the enforcement of this Agreement. The IDSA recognizes that the State of Connecticut's remedy at law regarding enforcement of this Agreement (including the Action Plan) is inadequate and agrees that the Connecticut Superior Court has the authority specifically to enforce the provisions of this Agreement, including the authority to award equitable relief. The exclusive forum for resolving any disputes under this Agreement shall be the superior court for the Judicial District of Hartford, or as otherwise required by law, and the IDSA consents to the jurisdiction of the State of Connecticut only for the purpose of an action by the Attorney General to enforce the terms of this Agreement. If compliance with any aspect of this Agreement proves impracticable, the IDSA may request that the Attorney General agree to modify the Agreement.

5. By entering into this Agreement – and by its prior actions cooperating with the Attorney General's Investigation – the IDSA has not consented to the jurisdiction of the State of Connecticut and has not waived its right to object on any ground to the power of the Attorney General (or any party) to bring any action against the IDSA in any forum, other than as stated in provision 4 above regarding enforcement of this Agreement.

6. This Agreement shall not confer any rights upon any persons or entities besides the Attorney General and the IDSA and shall be enforceable only by the parties to this Agreement.

7. The parties to this Agreement will use good faith and best efforts in implementing the terms of this Agreement (including the Action Plan) and in resolving any unanticipated issues.

8. In any application, legal action, or proceeding, email or facsimile transmission of any papers to current counsel for the IDSA shall be good and sufficient service on the IDSA unless the IDSA designates, in writing to the Attorney General, another person to receive service by facsimile transmission.

9. This Agreement shall be governed by the laws of the State of Connecticut without regard to conflict of laws principles.

10. This Agreement may be modified by mutual written agreement of the Parties.

11. IDSA represents that it has fully read and understands this Agreement, that it understands the legal consequences involved in signing the Agreement, and that there are no other representations or agreements not stated in writing herein.

12. IDSA represents and warrants that it is represented by legal counsel, that it is fully advised of its legal rights in this matter, and that the person signing below is fully authorized to act on its behalf.

13. This Agreement shall constitute the entire agreement between the Attorney General and the IDSA and shall supersede any prior communication, understanding, or agreement, whether written or oral, concerning the subject matter of this Agreement.

14. If any portion or part of this Agreement is held invalid, unenforceable, or void for any reason whatsoever, that portion shall be severed from the remainder of this Agreement and shall not affect the validity or enforceability of the remaining portions of this Agreement.

15. This Agreement may be executed in counterparts.

**WHEREFORE**, the following signatures are affixed hereto on this 30th day of April, 2008.

**STATE OF CONNECTICUT**

**RICHARD BLUMENTHAL**
**ATTORNEY GENERAL**

Michael E. Cole
Chief, Antitrust Department
Thomas P. Ryan
Assistant Attorneys General
Office of the Connecticut
Attorney General
55 Elm Street
PO Box 120
Hartford, CT 06141-0120
(860) 808-5040

**INFECTIOUS DISEASES SOCIETY OF AMERICA**

Mark Leasure
Executive Director
Infectious Diseases Society of America
1300 Wilson Blvd., Suite 300
Arlington, VA 22209
(703) 299-0200

14. If any portion or part of this Agreement is held invalid, unenforceable, or void for any reason whatsoever, that portion shall be severed from the remainder of this Agreement and shall not affect the validity or enforceability of the remaining portions of this Agreement.

15. This Agreement may be executed in counterparts.

**WHEREFORE**, the following signatures are affixed hereto on this 30th day of April, 2008.

**STATE OF CONNECTICUT**

**RICHARD BLUMENTHAL**
**ATTORNEY GENERAL**

______________________________

Michael E. Cole
Chief, Antitrust Department
Thomas P. Ryan
Assistant Attorneys General
Office of the Connecticut
Attorney General
55 Elm Street
PO Box 120
Hartford, CT 06141-0120
(860) 808-5040

**INFECTIOUS DISEASES SOCIETY OF AMERICA**

______________________________

Mark Leasure
Executive Director
Infectious Diseases Society of America
1300 Wilson Blvd., Suite 300
Arlington, VA 22209
(703) 299-0200

**EXHIBIT 1**

**Action Plan**

Infectious Diseases Society of America
Action Plan Requirements

I. The Infectious Diseases Society of America ("IDSA") shall convene a Review Panel whose task shall be to determine whether or not its 2006 Lyme disease guidelines, titled "The Clinical Assessment, Treatment, and Prevention of Lyme Disease, Human Granulocytic Anaplasmosis, and Babesiosis: Clinical Practice Guidelines by the Infectious Diseases Society of America," (hereinafter, "2006 Lyme disease guidelines") should be revised or updated based on a review of all relevant evidence, including any evidence submitted through this review process. A Review Panel of not less than eight but not more than twelve members, none of whom served on the 2006 Lyme disease guideline panel, shall be convened for this review. The Office of the Connecticut Attorney General ("CTOAG") and the IDSA shall jointly select an Ombudsman whose duties are set forth in this Action Plan.

The Review Panel will be formed and conduct its responsibilities as follows:

A. Review Panel Chairperson Selection:

1. Selection of the Review Panel Chairperson shall be made by the IDSA's Standards and Practice Guidelines Committee ("SPGC") through an open application process.
2. Applicants shall disclose all financial relationships and competing interests via the Applicant Statement of Interests that is attached hereto as Appendix 1. Following said disclosure, the SPGC shall select a Chairperson that it and the Ombudsman have determined to be without any beneficial or financial interest related to Lyme disease, any financial relationship with an entity that has an interest in Lyme disease, and any conflict of interest.[1]
3. In selecting a Chairperson for the Review Panel, the SPGC shall use the following criteria:
   a. Must be trained in infectious diseases.

---

1 A conflict of interest exists when anyone involved in the guideline process has a financial or other beneficial interest in the products or concepts addressed in the guidelines or in competing products or concepts that might bias his or her judgment. For guidance purposes, if the combined financial or beneficial interests in the products or concepts addressed in the guidelines exceed $10,000, those interests may be considered to bias a participant's judgment.

b. Must not have previously published a particular viewpoint regarding Lyme disease diagnosis or treatment.

c. Must be knowledgeable about the subject of Lyme disease, but not necessarily an expert.

d. Must have experience in the review and interpretation of the medical/scientific literature.

e. Must have known abilities to:

i. Complete tasks in a timely manner.

ii. Consider varying points of view.

iii. Bring groups of individuals to consensus.

f. Must not have served on any Lyme disease guideline panel.

B. Review Panelist Selection: A Review Panel of no fewer than eight but no more than twelve panelists (including the Chairperson, who shall be a full member of the Review Panel) shall be selected by the SPGC and the Chairperson.

1. Review Panelist applicants shall be solicited by a fair and open application process.

a. Applicants for Chairperson may be considered for inclusion in the Review Panel.

b. IDSA shall post an announcement on the IDSA website encouraging interested clinicians and/or scientists to apply.

c. Applications from representatives of other relevant specialties may also be solicited by the IDSA.

2. Applicants shall disclose all financial relationships and competing interests via the Applicant Statement of Interests that is attached hereto as Appendix 1. Following such disclosure, the SPGC and the Chairperson shall select Review Panelist applicants that the SPGC Chair and the Ombudsman have determined to be without any conflicts of interest.

3. The SPGC shall select Review Panelists who, as a group, reflect a balanced variety of perspectives and experience across a broad range of relevant disciplines, ranging from clinical experience in treating patients with Lyme disease to experience in investigating the best methods to diagnose and treat Lyme disease or other infectious diseases.

4. The Review Panel shall include at least one physician with clinical experience in treating Lyme disease patients.

5. Review Panelists need not be members of the IDSA.

6. The SPGC shall give fair consideration to all reasonable applicants.

7. Review Panelists shall not have previously served on any Lyme disease guideline panel.

C. Review Panel Operation:

1. Data Collection

a. Under the direction of the Review Panel, IDSA Staff shall conduct a comprehensive search and retrieval of the medical/scientific literature, which shall be considered by the Review Panel along with other literature submitted through the hearing or input collection mechanisms identified in subsections 1.b and 2.b of this section.

b. IDSA Staff shall post a conspicuous announcement of its intention to collect medical/scientific evidence related to Lyme disease on the IDSA website and shall develop an online mechanism, which shall include a dedicated e-mail address, to collect input from individuals and organizations that shall be disseminated to and considered by the Review Panel.

c. Input period shall be open for at least 60 days. Such period shall precede the Review Panel's commencement of its assessment of the 2006 Lyme disease guidelines.

2. Meetings

a. The Review Panel shall meet at least once in person and as needed via teleconference to consider all relevant evidence and all input submitted, as indicated above.

b. An open public hearing shall be held in conjunction with an in-person Review Panel meeting to offer a forum for the presentation of relevant written or oral data/information on the topic of Lyme disease. All public stakeholders may apply to make an oral presentation; however, clinicians and researchers shall be given preference. The Review Panel shall work with the Ombudsman and the CTOAG to finalize a list of presenters and shall reserve presentation time for divergent opinions. The presenters shall include a minimum of two members of the 2006 IDSA Lyme disease guideline panel. Individuals making presentations at the hearing shall disclose all conflicts of interest to the Review Panel by submitting a certified statement. A conflict of interest shall not be grounds for denial of the opportunity to present.

i. The IDSA shall conduct a live video broadcast of the hearing for public viewing on its website. All oral statements made during the

hearing shall be recorded in the official transcript. Such transcript and copies of all written information provided by the individuals making presentations shall be made part of the Review Panel record and shall be made available to the public.

3. Weighing the Evidence

   a. The principle function of the Review Panel shall be to make an individual determination whether each of the recommendations in the 2006 Lyme disease guidelines is medically/scientifically justified in light of all of the evidence and information provided.

   b. In evaluating the need for a revision or update, the Panel may consider the following questions (Shenkelle, *et al.*[2]):

      i. Has information about the magnitude of benefits and harms rendered the pre-existing guidelines invalid?

      ii. Has evidence identified important outcomes that need to be added to or considered by the guidelines (e.g., quality of life)?

      iii. Are there preventive, diagnostic, or treatment interventions to complement or supersede the interventions in the pre-existing guidelines?

      iv. Does the evidence show that current practice is optimal and the guidelines are no longer needed?

      v. Have there been changes in the values placed on outcomes?

      vi. Have there been changes in the resources available in healthcare (e.g., availability of less expensive (generic) drugs)?

4. Voting

   a. The Review Panel shall strive to achieve consensus.

   b. It is the responsibility of the Review Panel Chairperson to manage any vote on any key finding or recommendation and report such vote to the Ombudsman. The name and vote of each Review Panel member must be maintained for the record, but will not be made public, though the overall vote of the Review Panel on the final recommendation(s) shall be made public.

---

[2] Shenkelle P, Eccles MP, Grimshaw JM, Woolf SH: When should clinical guidelines be updated? BMJ 2001;323;155-157.4

c. Panel determinations/recommendations shall require a supermajority vote of 75% or more of the total voting members.

5. Recommendation

a. Based on its weighing of evidence, the Review Panel shall recommend one of the following three options:

i. That no changes to the 2006 Lyme disease guidelines are necessary.

ii. That there is a need for sectional revision of the 2006 Lyme disease guideline. In this instance the Review Panel shall make proposals for those revisions, which shall be considered and implemented by the SPGC.

iii. That a complete rewriting of the 2006 Lyme disease guideline is required. If the Review Panel determines that such a rewriting of the Lyme disease guideline is warranted, the IDSA shall convene a guideline panel consistent with the terms of this Action Plan and the and the IDSA's Handbook on Clinical Practice Guideline Development.

6. The recommendation(s) of the Review Panel shall be binding upon the IDSA.

D. Final Report:

1. The Final Report shall be certified by the Review Panel Chairperson and shall include the following:

a. The names of each Review Panelist.

b. Statements of whether each recommendation in the 2006 Lyme disease guidelines was found by the Review Panel to be medically/scientifically justified in light of the evidence and information collected and provided.

c. A statement of the Review Panel's overall recommendation pursuant to subsection C.5 of this section, including any particular recommended revisions pursuant to C.5.a.ii.

2. The IDSA shall conspicuously place a link to the Final Report on its website's home page for one year following the release of the Final Report.[3] The IDSA shall also provide copies of the Final Report to any organization that endorsed

---

[3] Should the Review Panel recommend a sectional revision or complete rewriting of the 2006 Lyme disease guideline pursuant to subsection C.5, then the IDSA shall continue to place a link on its website's homepage until such time as such revision or rewrite is complete.

the 2006 Lyme disease guidelines, the National Guidelines Clearinghouse, and the CTOAG.

E. Records and Minutes of Meetings:

1. IDSA shall retain all records relating to the Review Panel's activities, including the selection of the Review Panel Chairperson and Review Panelists. All vote tallies shall be recorded.

2. Official minutes of all in-person and telephonic panel meetings shall be recorded and maintained.

3. The Ombudsman and CTOAG shall have access to all records and minutes of all Review Panel meetings. IDSA shall provide copies of all records and minutes of Review Panel meetings to the Ombudsman, who shall keep such records confidential with respect to persons who are not parties to this Agreement. In the event that the CTOAG requires access to such documents, IDSA shall make them available for inspection and review at its offices and the office of its legal counsel in Connecticut.

## APPENDIX 1

Applicant Statement of Interests
(Financial, Equity, Intellectual Property, Research, Advocacy)

Name: Date of Statement: ____

Use and reference additional pages if necessary to complete this form.

1. PAST OR PRESENT FINANCIAL RELATIONSHIPS: Please list below all pharmaceutical, medical device, biotechnology, or medical consulting companies in which you or your immediate family member(s) have or have had financial, equity, or intellectual property interests, currently and in the 2 years prior to the date of this document.

| Name of Company | Type of Relationship (Please check (√) if yours or write "FM" if family member, defined as spouse and minor children) | | |
|---|---|---|---|
| | Financial* | Equity** | Intellectual Property |
| For interests ≤ $10,000 | | | |
| 1. | | | |
| 2. | | | |
| 3. | | | |
| 4. | | | |
| 5. | | | |
| 6. | | | |
| 7. | | | |
| 8. | | | |
| For interests > $10,000 to $25,000 | | | |
| 1. | | | |
| 2. | | | |
| 3. | | | |
| For interests > $25,000 | | | |
| 1. | | | |
| 2. | | | |
| 3. | | | |

*Fees for consulting, speaker's bureaus, advisory boards, or other committees. Include fees paid to you directly or indirectly to you through a University account that is under your control (e.g., discretionary account).
**Do NOT include mutual funds.

400810128v3

2. FUTURE STOCK OPTIONS/PATENT RIGHTS: Please list all stock options and/or patent rights that you or your family member(s) have in a pharmaceutical, medical device, or biotechnology company. Include pertinent patent numbers.

| Name of Company | Type of INTEREST (Please check (√) if yours or write "FM" if family member) | |
|---|---|---|
| | Future Stock Options | Patent Rights |
| 1. | | |
| 2. | | |
| 3. | | |
| 4. | | |
| 5. | | |
| 6. | | |
| 7 | | |
| 8. | | |

3. List any grant or contract that either provides salary support paid to you through your institution or supports your research without salary support, currently and in the 2 years prior to the date of this document. Only include research that could reasonably be considered related to Lyme disease.

| Name of Sponsor* | Brief Description of Research |
|---|---|
| 1. | |
| 2. | |
| 3. | |
| 4. | |
| 5. | |
| 6. | |
| 7. | |

*List Government (e.g., NIH, FDA, AHRQ), Foundation source, name of private company (e.g., pharmaceutical, medical device, biotechnology, or medical consulting company), name of individual, or name of partnership, trust, or any other entity.

4. List all medical contracts not disclosed above, excluding contracts for the direct provision of medical care to patients, but including insurance and medical consulting contracts.

| Name of Contractor | Description of Contract |
|---|---|
| 1. | |
| 2. | |
| 3. | |
| 4. | |
| 5. | |
| 6. | |
| 7. | |

5. Estimate the percentage of your clinical practice that is devoted to the diagnoses and treatment of patients for Lyme disease.

_____%

Estimate the amount of revenue generated by your clinical services to diagnose and treat patients for Lyme disease.

☐ <$10,000

☐ >$10,000-$25, 000

☐ >$25,000

6. In the past 2 years, did you serve as an owner, officer, director, partner, manager, or employee of any pharmaceutical, medical device, or biotechnology company?
No ______ Yes ______ If yes, specify the company(s) and details of your role.

7. In the past 2 years, have you received payment for expert testimony in a legal proceeding on a topic that could reasonably be considered related to Lyme disease?
No ______ Yes ______ If yes, specify content area of your testimony.

8. In the past 2 years, have you received payment for an advocacy role in government or non-profit organization on a topic that could reasonably be considered related to Lyme disease?
No ______ Yes ______ If yes, specify advocacy role.

**CERTIFICATION**

State of _________________)
                                          )        ss:
County of ________________)

I ______________________________hereby certify that the information provided above is true, accurate and complete to the best of my knowledge and belief.

Dated: ________________, 20___

________________________________          ___________________________
Print Name                                                    Signature

Subscribed to and sworn before me this ________________day of

__________________20___.

________________________________
Notary Public

My commission expires _____________, 20____
