# Supplementary material for: The Infectious Diseases Society of America Lyme guidelines: a cautionary tale about the development of clinical practice guidelines
Source: Philos Ethics Humanit Med. 2010 Jun 9;5:9. doi: 10.1186/1747-5341-5-9 (PMC2901226; doi:10.1186/1747-5341-5-9)
Supplement: Additional file 2 — CT AG Press Release re: IDSA Settlement Agreement. Press Release of Connecticut Attorney General dated May 1, 2008 regarding settlement with the IDSA of antitrust investigation and findings of investigation. [file 1747-5341-5-9-S2.PDF]

Office of the Attorney General
55 Elm Street
Hartford, Connecticut
06106

Telephone:
(860) 808-5318

Connecticut Attorney General's Office

Press Release

Attorney General's Investigation Reveals Flawed Lyme Disease Guideline Process, IDSA Agrees To Reassess Guidelines, Install Independent Arbiter

May 1, 2008

Attorney General Richard Blumenthal today announced that his antitrust investigation has uncovered serious flaws in the Infectious Diseases Society of America's (IDSA) process for writing its 2006 Lyme disease guidelines and the IDSA has agreed to reassess them with the assistance of an outside arbiter.

The IDSA guidelines have sweeping and significant impacts on Lyme disease medical care. They are commonly applied by insurance companies in restricting coverage for long-term antibiotic treatment or other medical care and also strongly influence physician treatment decisions.

Insurance companies have denied coverage for long-term antibiotic treatment relying on these guidelines as justification. The guidelines are also widely cited for conclusions that chronic Lyme disease is nonexistent.

"This agreement vindicates my investigation -- finding undisclosed financial interests and forcing a reassessment of IDSA guidelines," Blumenthal said. "My office uncovered undisclosed financial interests held by several of the most powerful IDSA panelists. The IDSA's guideline panel improperly ignored or minimized consideration of alternative medical opinion and evidence regarding chronic Lyme disease, potentially raising serious questions about whether the recommendations reflected all relevant science.

"The IDSA's Lyme guideline process lacked important procedural safeguards requiring complete reevaluation of the 2006 Lyme disease guidelines -- in effect a comprehensive reassessment through a new panel. The new panel will accept and analyze all evidence, including divergent opinion. An independent neutral ombudsman -- expert in medical ethics and conflicts of interest, selected by both the IDSA and my office -- will assess the new panel for conflicts of interests and ensure its integrity."

Blumenthal's findings include the following:

- The IDSA failed to conduct a conflicts of interest review for any of the panelists prior to their appointment to the 2006 Lyme disease guideline panel;
- Subsequent disclosures demonstrate that several of the 2006 Lyme disease panelists had conflicts of interest;
- The IDSA failed to follow its own procedures for appointing the 2006 panel chairman and members, enabling the chairman, who held a bias regarding the existence of chronic Lyme, to handpick a likeminded panel without scrutiny by or formal approval of the IDSA's oversight committee;
- The IDSA's 2000 and 2006 Lyme disease panels refused to accept or meaningfully consider information regarding the existence of chronic Lyme disease, once removing a panelist from the 2000 panel who dissented from the group's position on chronic Lyme disease to achieve "consensus";
- The IDSA blocked appointment of scientists and physicians with divergent views on chronic Lyme who sought to serve on the 2006 guidelines panel by informing them that the panel was fully staffed, even though it was later expanded;
- The IDSA portrayed another medical association's Lyme disease guidelines as corroborating its own when it knew that the two panels shared several authors, including the chairmen of both groups, and were working on guidelines at the same time. In allowing its panelists to serve on both groups at the same time, IDSA violated its own conflicts of interest policy.

IDSA has reached an agreement with Blumenthal's office calling for creation of a

review panel to thoroughly scrutinize the 2006 Lyme disease guidelines and update or revise them if necessary. The panel -- comprised of individuals without conflicts of interest -- will comprehensively review medical and scientific evidence and hold a scientific hearing to provide a forum for additional evidence. It will then determine whether each recommendation in the 2006 Lyme disease guidelines is justified by the evidence or needs revision or updating.

Blumenthal added, "The IDSA's 2006 Lyme disease guideline panel undercut its credibility by allowing individuals with financial interests -- in drug companies, Lyme disease diagnostic tests, patents and consulting arrangements with insurance companies -- to exclude divergent medical evidence and opinion. In today's healthcare system, clinical practice guidelines have tremendous influence on the marketing of medical services and products, insurance reimbursements and treatment decisions. As a result, medical societies that publish such guidelines have a legal and moral duty to use exacting safeguards and scientific standards.

"Our investigation was always about the IDSA's guidelines process -- not the science. IDSA should be recognized for its cooperation and agreement to address the serious concerns raised by my office. Our agreement with IDSA ensures that a new, conflicts-free panel will collect and review all pertinent information, reassess each recommendation and make necessary changes.

"This Action Plan -- incorporating a conflicts screen by an independent neutral expert and a public hearing to receive additional evidence -- can serve as a model for all medical organizations and societies that publish medical guidelines. This review should strengthen the public's confidence in such critical standards."

THE GUIDELINE REVIEW PROCESS

Under its agreement with the Attorney General's Office, the IDSA will create a review panel of eight to 12 members, none of whom served on the 2006 IDSA guideline panel. The IDSA must conduct an open application process and consider all applicants.

The agreement calls for the ombudsman selected by Blumenthal's office and the IDSA to ensure that the review panel and its chairperson are free of conflicts of interest.

Blumenthal and IDSA agreed to appoint Dr. Howard A. Brody as the ombudsman. Dr. Brody is a recognized expert and author on medical ethics and conflicts of interest and the director of the Institute for Medical Humanities at the University of Texas Medical Branch. Brody authored the book, "Hooked: Ethics, the Medical Profession and the Pharmaceutical Industry."

To assure that the review panel obtains divergent information, the panel will conduct an open scientific hearing at which it will hear scientific and medical presentations from interested parties. The agreement requires the hearing to be broadcast live to the public on the Internet via the IDSA's website. The Attorney General's Office, Dr. Brody and the review panel will together finalize the list of presenters at the hearing.

Once it has collected information from its review and open hearing, the panel will assess the information and determine whether the data and evidence supports each of the recommendations in the 2006 Lyme disease guidelines.

The panel will then vote on each recommendation in the IDSA's 2006 Lyme disease guidelines on whether it is supported by the scientific evidence. At least 75 percent of panel members must vote to sustain each recommendation or it will be revised.

Once the panel has acted on each recommendation, it will have three options: make no changes, modify the guidelines in part or replace them entirely.

The panel's final report will be published on the IDSA's website.

ADDITIONAL FINDINGS OF BLUMENTHAL'S INVESTIGATION

IDSA convened panels in 2000 and 2006 to research and publish guidelines for the diagnosis and treatment of Lyme disease. Blumenthal's office found that the IDSA disregarded a 2000 panel member who argued that chronic and persistent Lyme disease exists. The 2000 panel pressured the panelist to conform to the group consensus and removed him as an author when he refused.

IDSA sought to portray a second set of Lyme disease guidelines issued by the American Academy of Neurology (AAN) as independently corroborating its findings. In fact, IDSA knew that the two panels shared key members, including the respective panel chairmen and were working on both sets of guidelines a the same time -- a violation of IDSA's conflicts of interest policy.

The resulting IDSA and AAN guidelines not only reached the same conclusions

regarding the non-existence of chronic Lyme disease, their reasoning at times used strikingly similar language. Both entities, for example, dubbed symptoms persisting after treatment "Post-Lyme Syndrome" and defined it the same way.

When IDSA learned of the improper links between its panel and the AAN's panel, instead of enforcing its conflict of interest policy, it aggressively sought the AAN's endorsement to "strengthen" its guidelines' impact. The AAN panel -- particularly members who also served on the IDSA panel -- worked equally hard to win AAN's backing of IDSA's conclusions.

The two entities sought to portray each other's guidelines as separate and independent when the facts call into question that contention.

The IDSA subsequently cited AAN's supposed independent corroboration of its findings as part of its attempts to defeat federal legislation to create a Lyme disease advisory committee and state legislation supporting antibiotic therapy for chronic Lyme disease.

In a step that the British Medical Journal deemed "unusual," the IDSA included in its Lyme guidelines a statement calling them "voluntary" with "the ultimate determination of their application to be made by the physician in light of each patient's individual circumstances." In fact, United Healthcare, Health Net, Blue Cross of California, Kaiser Foundation Health Plan and other insurers have used the guidelines as justification to deny reimbursement for long-term antibiotic treatment.

Blumenthal thanked members his office who worked on the investigation -- Assistant Attorney General Thomas Ryan, former Assistant Attorney General Steven Rutstein and Paralegal Lorraine Measer under the direction of Assistant Attorney General Michael Cole, Chief of the Attorney General's Antitrust Department.

View the entire IDSA agreement - (PDF-2,532KB)

Content Last Modified on 5/14/2008 8:40:39 AM

Printable Version

State of Connecticut Disclaimer and Privacy Policy Copyright © 2002 - 2010 State of Connecticut
