# Supplementary material for: The Infectious Diseases Society of America Lyme guidelines: a cautionary tale about the development of clinical practice guidelines
Source: Philos Ethics Humanit Med. 2010 Jun 9;5:9. doi: 10.1186/1747-5341-5-9 (PMC2901226; doi:10.1186/1747-5341-5-9)
Supplement: Additional file 3 — IDSA internal memo re: voting and action plan. Internal memo dated July 9, 2009 from Jennifer Padberg to IDSA Lyme guidelines review panel regarding voting procedure requirements. [file 1747-5341-5-9-S3.PDF]

IDSA

Infectious Diseases Society of America

2008-2009
BOARD OF DIRECTORS

President
Anne A. Gershon, MD, FIDSA
Columbia University College of Physicians
New York, NY

President-Elect
Richard J. Whitley, MD, FIDSA
University of Alabama at Birmingham
Birmingham, AL

Vice President
James M. Hughes, MD, FIDSA
Emory University
Atlanta, GA

Secretary
William Schaffner, MD, FIDSA
Vanderbilt University School of Medicine
Nashville, TN

Treasurer
Barbara E. Murray, MD, FIDSA
University of Texas Medical School
Houston, TX

Immediate Past President
Donald M. Poretz, MD, FIDSA
Infectious Diseases Physicians
Annandale, VA

Stephen B. Calderwood, MD, FIDSA
Massachusetts General Hospital
Boston, MA

Thomas M. File, MD, FIDSA
Summa Health System
Akron, OH

Carol A. Kauffman, MD, FIDSA
University of Michigan Medical School
Ann Arbor, MI

Sandra A. Kemmerly, MD, FIDSA
Ochsner Health System
New Orleans, LA

Daniel R. Kuritzkes, MD, FIDSA
Brigham and Women's Hospital
Boston, MA

Jan E. Patterson, MD, FIDSA
University of Texas Health Science Center
San Antonio, TX

William G. Powderly, MD, FIDSA
University College Dublin
Dublin, Ireland

Edward J. Septimus, MD, FIDSA
HCA Healthcare System
Houston, TX

Robert A. Weinstein, MD, FIDSA
John Stroger Hospital of Cook County
Chicago, IL

Chief Executive Officer
Mark A. Leasure

IDSA Headquarters
1300 Wilson Boulevard
Suite 300
Arlington, VA 22209
TEL: (703) 299-0200
FAX: (703) 299-0204
EMAIL ADDRESS:
info@idsociety.org
WEBSITE:
www.idsociety.org

July 9, 2009

To: IDSA Lyme Disease Guideline Review Panel

From: Jennifer J. Padberg, MPH

Re: To revisit the Panels' overall purpose and goals

Sent via e-mail

Dear Review Panel Members:

We are quickly approaching the July 30-31, hearing and panel meeting and as we discussed during our conference call in June, a reiteration of the purpose and goals of this effort may be helpful to you as we move into the events taking place in just a few short weeks. I have discussed these purposes and goals with the Connecticut Attorney General's Office. We agree that under the Action Plan, a copy of which is attached hereto for your review, the Review Panel must do the following:

- Objectively review and consider all the relevant evidence, including the information gathered (literature, public input) and presented (at the public hearing);
    - o Evidence published after the publication of the 2006 guidelines should also be considered.
- The principle function of the Review Panel shall be to make an individual determination whether each of the recommendations in the IDSA's 2006 Lyme disease guidelines is medically/scientifically justified in light of all of the evidence and information provided.
    - o Each Panel member must vote on each recommendation within the 2006 Guidelines
    - o Upon completion of voting on the individual recommendations in the 2006 guidelines, each Panel member must vote on an overall recommendation for the guidelines as follows:
        - ▪ No changes are necessary, OR
        - ▪ Sectional revision is needed; proposals for any such revision(s) should be made, OR
        - ▪ Complete re-writing is needed
    - o All votes require supermajority support (75% or more). Hence a minimum of seven panel members must vote in favor of a recommendation in order for the panel to deem it supported by the

evidence, just as a minimum of seven panel members must support one of the three options for the overall guideline evaluation in order to recommend that option to the IDSA.
    - All voting will be recorded by the Chaiperson and presented to the ombudsman.
- The Panel's overall recommendation and any proposed revisions must be submitted to the IDSA.
- A final report will be developed and made publically available.

We thank you for your committement of time and effort to this important process and for your anticipated objectivity in reviewing the evidence presented. If you have any questions about this information, please let me know (phone: 703/299-0162; e-mail: jpadberg@idsociety.org).

Sincerely,

Jennifer J. Padberg

Jennifer J. Padberg, MPH
Vice President of Clinical Affairs

cc: Richard Blumenthal, Attorney General, State of Connecticut
Howard Brody, MD, Ombudsman
Thomas Ryan, Assistant Attorney General, CT Office of the Attorney General

attachment

EXHIBIT 1

Action Plan

Infectious Diseases Society of America
Action Plan Requirements

I. The Infectious Diseases Society of America ("IDSA") shall convene a Review Panel whose task shall be to determine whether or not its 2006 Lyme disease guidelines, titled "The Clinical Assessment, Treatment, and Prevention of Lyme Disease, Human Granulocytic Anaplasmosis, and Babesiosis: Clinical Practice Guidelines by the Infectious Diseases Society of America," (hereinafter, "2006 Lyme disease guidelines") should be revised or updated based on a review of all relevant evidence, including any evidence submitted through this review process. A Review Panel of not less than eight but not more than twelve members, none of whom served on the 2006 Lyme disease guideline panel, shall be convened for this review. The Office of the Connecticut Attorney General ("CTOAG") and the IDSA shall jointly select an Ombudsman whose duties are set forth in this Action Plan.

The Review Panel will be formed and conduct its responsibilities as follows:

A. Review Panel Chairperson Selection:

1. Selection of the Review Panel Chairperson shall be made by the IDSA's Standards and Practice Guidelines Committee ("SPGC") through an open application process.
2. Applicants shall disclose all financial relationships and competing interests via the Applicant Statement of Interests that is attached hereto as Appendix 1. Following said disclosure, the SPGC shall select a Chairperson that it and the Ombudsman have determined to be without any beneficial or financial interest related to Lyme disease, any financial relationship with an entity that has an interest in Lyme disease, and any conflict of interest.[1]
3. In selecting a Chairperson for the Review Panel, the SPGC shall use the following criteria:
   a. Must be trained in infectious diseases.

---

1 A conflict of interest exists when anyone involved in the guideline process has a financial or other beneficial interest in the products or concepts addressed in the guidelines or in competing products or concepts that might bias his or her judgment. For guidance purposes, if the combined financial or beneficial interests in the products or concepts addressed in the guidelines exceed $10,000, those interests may be considered to bias a participant's judgment.

b. Must not have previously published a particular viewpoint regarding Lyme disease diagnosis or treatment.

c. Must be knowledgeable about the subject of Lyme disease, but not necessarily an expert.

d. Must have experience in the review and interpretation of the medical/scientific literature.

e. Must have known abilities to:

i. Complete tasks in a timely manner.

ii. Consider varying points of view.

iii. Bring groups of individuals to consensus.

f. Must not have served on any Lyme disease guideline panel.

B. Review Panelist Selection: A Review Panel of no fewer than eight but no more than twelve panelists (including the Chairperson, who shall be a full member of the Review Panel) shall be selected by the SPGC and the Chairperson.

1. Review Panelist applicants shall be solicited by a fair and open application process.

a. Applicants for Chairperson may be considered for inclusion in the Review Panel.

b. IDSA shall post an announcement on the IDSA website encouraging interested clinicians and/or scientists to apply.

c. Applications from representatives of other relevant specialties may also be solicited by the IDSA.

2. Applicants shall disclose all financial relationships and competing interests via the Applicant Statement of Interests that is attached hereto as Appendix 1. Following such disclosure, the SPGC and the Chairperson shall select Review Panelist applicants that the SPGC Chair and the Ombudsman have determined to be without any conflicts of interest.

3. The SPGC shall select Review Panelists who, as a group, reflect a balanced variety of perspectives and experience across a broad range of relevant disciplines, ranging from clinical experience in treating patients with Lyme disease to experience in investigating the best methods to diagnose and treat Lyme disease or other infectious diseases.

4. The Review Panel shall include at least one physician with clinical experience in treating Lyme disease patients.

5. Review Panelists need not be members of the IDSA.

6. The SPGC shall give fair consideration to all reasonable applicants.

7. Review Panelists shall not have previously served on any Lyme disease guideline panel.

C. Review Panel Operation:

1. Data Collection

a. Under the direction of the Review Panel, IDSA Staff shall conduct a comprehensive search and retrieval of the medical/scientific literature, which shall be considered by the Review Panel along with other literature submitted through the hearing or input collection mechanisms identified in subsections 1.b and 2.b of this section.

b. IDSA Staff shall post a conspicuous announcement of its intention to collect medical/scientific evidence related to Lyme disease on the IDSA website and shall develop an online mechanism, which shall include a dedicated e-mail address, to collect input from individuals and organizations that shall be disseminated to and considered by the Review Panel.

c. Input period shall be open for at least 60 days. Such period shall precede the Review Panel's commencement of its assessment of the 2006 Lyme disease guidelines.

2. Meetings

a. The Review Panel shall meet at least once in person and as needed via teleconference to consider all relevant evidence and all input submitted, as indicated above.

b. An open public hearing shall be held in conjunction with an in-person Review Panel meeting to offer a forum for the presentation of relevant written or oral data/information on the topic of Lyme disease. All public stakeholders may apply to make an oral presentation; however, clinicians and researchers shall be given preference. The Review Panel shall work with the Ombudsman and the CTOAG to finalize a list of presenters and shall reserve presentation time for divergent opinions. The presenters shall include a minimum of two members of the 2006 IDSA Lyme disease guideline panel. Individuals making presentations at the hearing shall disclose all conflicts of interest to the Review Panel by submitting a certified statement. A conflict of interest shall not be grounds for denial of the opportunity to present.

i. The IDSA shall conduct a live video broadcast of the hearing for public viewing on its website. All oral statements made during the

hearing shall be recorded in the official transcript. Such transcript and copies of all written information provided by the individuals making presentations shall be made part of the Review Panel record and shall be made available to the public.

3. Weighing the Evidence

   a. The principle function of the Review Panel shall be to make an individual determination whether each of the recommendations in the 2006 Lyme disease guidelines is medically/scientifically justified in light of all of the evidence and information provided.

   b. In evaluating the need for a revision or update, the Panel may consider the following questions (Shenkelle, *et al.*[2]):

      i. Has information about the magnitude of benefits and harms rendered the pre-existing guidelines invalid?

      ii. Has evidence identified important outcomes that need to be added to or considered by the guidelines (e.g., quality of life)?

      iii. Are there preventive, diagnostic, or treatment interventions to complement or supersede the interventions in the pre-existing guidelines?

      iv. Does the evidence show that current practice is optimal and the guidelines are no longer needed?

      v. Have there been changes in the values placed on outcomes?

      vi. Have there been changes in the resources available in healthcare (e.g., availability of less expensive (generic) drugs)?

4. Voting

   a. The Review Panel shall strive to achieve consensus.

   b. It is the responsibility of the Review Panel Chairperson to manage any vote on any key finding or recommendation and report such vote to the Ombudsman. The name and vote of each Review Panel member must be maintained for the record, but will not be made public, though the overall vote of the Review Panel on the final recommendation(s) shall be made public.

---

2 Shenkelle P, Eccles MP, Grimshaw JM, Woolf SH: When should clinical guidelines be updated? BMJ 2001;323;155-157.4

c. Panel determinations/recommendations shall require a supermajority vote of 75% or more of the total voting members.

5. Recommendation

a. Based on its weighing of evidence, the Review Panel shall recommend one of the following three options:

i. That no changes to the 2006 Lyme disease guidelines are necessary.

ii. That there is a need for sectional revision of the 2006 Lyme disease guideline. In this instance the Review Panel shall make proposals for those revisions, which shall be considered and implemented by the SPGC.

iii. That a complete rewriting of the 2006 Lyme disease guideline is required. If the Review Panel determines that such a rewriting of the Lyme disease guideline is warranted, the IDSA shall convene a guideline panel consistent with the terms of this Action Plan and the and the IDSA's Handbook on Clinical Practice Guideline Development.

6. The recommendation(s) of the Review Panel shall be binding upon the IDSA.

D. Final Report:

1. The Final Report shall be certified by the Review Panel Chairperson and shall include the following:

a. The names of each Review Panelist.

b. Statements of whether each recommendation in the 2006 Lyme disease guidelines was found by the Review Panel to be medically/scientifically justified in light of the evidence and information collected and provided.

c. A statement of the Review Panel's overall recommendation pursuant to subsection C.5 of this section, including any particular recommended revisions pursuant to C.5.a.ii.

2. The IDSA shall conspicuously place a link to the Final Report on its website's home page for one year following the release of the Final Report.[3] The IDSA shall also provide copies of the Final Report to any organization that endorsed

---

3 Should the Review Panel recommend a sectional revision or complete rewriting of the 2006 Lyme disease guideline pursuant to subsection C.5, then the IDSA shall continue to place a link on its website's homepage until such time as such revision or rewrite is complete.

the 2006 Lyme disease guidelines, the National Guidelines Clearinghouse, and the CTOAG.

E. Records and Minutes of Meetings:

1. IDSA shall retain all records relating to the Review Panel's activities, including the selection of the Review Panel Chairperson and Review Panelists. All vote tallies shall be recorded.

2. Official minutes of all in-person and telephonic panel meetings shall be recorded and maintained.

3. The Ombudsman and CTOAG shall have access to all records and minutes of all Review Panel meetings. IDSA shall provide copies of all records and minutes of Review Panel meetings to the Ombudsman, who shall keep such records confidential with respect to persons who are not parties to this Agreement. In the event that the CTOAG requires access to such documents, IDSA shall make them available for inspection and review at its offices and the office of its legal counsel in Connecticut.

APPENDIX 1

Applicant Statement of Interests
(Financial, Equity, Intellectual Property, Research, Advocacy)

Name: Date of Statement: ____

Use and reference additional pages if necessary to complete this form.

1. PAST OR PRESENT FINANCIAL RELATIONSHIPS: Please list below all pharmaceutical, medical device, biotechnology, or medical consulting companies in which you or your immediate family member(s) have or have had financial, equity, or intellectual property interests, currently and in the 2 years prior to the date of this document.

Name of Company	Type of Relationship (Please check (√) if yours or write "FM" if family member, defined as spouse and minor children)		
	Financial*	Equity**	Intellectual Property
For interests ≤ $10,000			
1.			
2.			
3.			
4.			
5.			
6.			
7.			
8.			
For interests > $10,000 to $25,000			
1.			
2.			
3.			
For interests > $25,000			
1.			
2.			
3.			

*Fees for consulting, speaker's bureaus, advisory boards, or other committees. Include fees paid to you directly or indirectly to you through a University account that is under your control (e.g., discretionary account).
**Do NOT include mutual funds.

2. FUTURE STOCK OPTIONS/PATENT RIGHTS: Please list all stock options and/or patent rights that you or your family member(s) have in a pharmaceutical, medical device, or biotechnology company. Include pertinent patent numbers.

Name of Company	Type of INTEREST (Please check (√) if yours or write "FM" if family member)	
	Future Stock Options	Patent Rights
1.		
2.		
3.		
4.		
5.		
6.		
7		
8.		

3. List any grant or contract that either provides salary support paid to you through your institution or supports your research without salary support, currently and in the 2 years prior to the date of this document. Only include research that could reasonably be considered related to Lyme disease.

Name of Sponsor*	Brief Description of Research
1.	
2.	
3.	
4.	
5.	
6.	
7.	

*List Government (e.g., NIH, FDA, AHRQ), Foundation source, name of private company (e.g., pharmaceutical, medical device, biotechnology, or medical consulting company), name of individual, or name of partnership, trust, or any other entity.

4. List all medical contracts not disclosed above, excluding contracts for the direct provision of medical care to patients, but including insurance and medical consulting contracts.

Name of Contractor	Description of Contract
1.	
2.	
3.	
4.	
5.	
6.	
7.	

5. Estimate the percentage of your clinical practice that is devoted to the diagnoses and treatment of patients for Lyme disease.

_____%

Estimate the amount of revenue generated by your clinical services to diagnose and treat patients for Lyme disease.

☐ <$10,000

☐ >$10,000-$25, 000

☐ >$25,000

6. In the past 2 years, did you serve as an owner, officer, director, partner, manager, or employee of any pharmaceutical, medical device, or biotechnology company?
No ______ Yes ______ If yes, specify the company(s) and details of your role.

7. In the past 2 years, have you received payment for expert testimony in a legal proceeding on a topic that could reasonably be considered related to Lyme disease?
No ______ Yes ______ If yes, specify content area of your testimony.

8. In the past 2 years, have you received payment for an advocacy role in government or non-profit organization on a topic that could reasonably be considered related to Lyme disease?
No ______ Yes ______ If yes, specify advocacy role.

CERTIFICATION

State of __________________)
)   ss:
County of ________________)

I ______________________________hereby certify that the information provided above is true, accurate and complete to the best of my knowledge and belief.

Dated: ________________, 20___

________________________________   ___________________________
Print Name   Signature

Subscribed to and sworn before me this _________________day of

__________________20___.

____
Notary Public

My commission expires _____________, 20____
