# Supplementary material for: The Infectious Diseases Society of America Lyme guidelines: a cautionary tale about the development of clinical practice guidelines
Source: Philos Ethics Humanit Med. 2010 Jun 9;5:9. doi: 10.1186/1747-5341-5-9 (PMC2901226; doi:10.1186/1747-5341-5-9)
Supplement: Additional file 4 — Letter from CT AG re: panel voting. Letter from Connecticut Attorney General dated February 1, 2010 to Richard Whitley, President of IDSA, regarding voting irregularities on IDSA Lyme guidelines review panel. [file 1747-5341-5-9-S4.PDF]

# State of Connecticut

RICHARD BLUMENTHAL
ATTORNEY GENERAL

Hartford

February 1, 2010

Richard J. Whitley, MD, FIDSA,
Infectious Disease Society of America
1300 Wilson Boulevard
Suite 300
Arlington, VA 22209

Dear Dr. Whitley:

I am writing to express my concern that the Infectious Diseases Society of America ("IDSA") may soon approve an improper voting procedure implemented by the Lyme disease Review Panel and its Chairperson in violation of the settlement agreement and Action Plan ("AP") with my office.

As you are aware, during an antitrust investigation of the IDSA my office uncovered significant procedural deficiencies relative to the IDSA's 2006 Lyme disease clinical practice guidelines. These process deficiencies raised serious questions as to whether the recommendations made in the 2006 guidelines reflected the best available scientific and medical evidence on the subject. In order to address the deficiencies uncovered during the investigation, my office and the IDSA negotiated a settlement, which included an AP. The essence of the AP was fairly simple: Step 1 to establish a stringent procedure for identifying and vetting a completely new review panel whose responsibility would be to, Step 2: assess by voting whether each of the original 2006 guidelines' recommendations are medically and scientifically justified in light of all the available evidence collected through an open collection process, and Step 3: through a vote, determine whether to make revisions to any of those recommendations or recommend a complete re-write of the 2006 guidelines.

Because my office determined that the IDSA had excluded consideration of divergent evidence and participation of individuals from the 2006 guidelines panel who held dissenting opinions, the cornerstone of the AP and the "principle function" of the Review Panel established thereunder, was to look anew and "make an individual determination whether each of the recommendations in the 2006 Lyme disease guidelines is medically/scientifically justified in

light of all of the evidence and information provided."[1]. In other words, the Review Panel was assigned the task of determining whether the Lyme disease panel "got it right" in the first instance for each recommendation.

In order to ensure there were no misunderstandings in the interpretation of the AP, attorneys from my office participated in a telephone call with the IDSA's attorneys on May 5, 2008, during which it was agreed that there were two voting elements in the agreement, each of which would require a supermajority confirmation. The first voting required, as part of the Review Panel's "weighing of the evidence responsibility", involves the "principal function" of the panel, which is "to make an individual determination whether each of the recommendations in the *2006 Lyme disease guidelines* is medically/scientifically justified in light of all of the evidence and information provided." [2] As our attorneys discussed, the term "determination" was specifically used in this section to ensure that votes be held on this question for each recommendation and that a supermajority be required to find that a particular recommendation was "medically/scientifically justified in light of all of the evidence and information provided." All "determinations/recommendations" require "a supermajority vote of 75% or more of total voting members."[3] Moreover, it was agreed that the Final Report requires disclosure of the outcome of this basic, yet fundamental voting element. In Section D.1(b), the AP requires that the report include "[s]tatements whether each recommendation in the 2006 Lyme disease guidelines was found by the Review Panel to be medically/scientifically justified in light of the evidence and information collected and provided" to demonstrate the outcome of this primary assessment. This primary voting was intended to elicit an affirmative yes/no results.

In anticipation of the Review Panel's issuance of the Final Report, members of my staff recently visited the office of your local counsel to view minutes and records pertaining to the Review Panel's voting. During this review my staff discovered that the Review Panel failed to conduct the principal voting required by the agreement and the AP on whether each recommendation in the 2006 Lyme disease guidelines was justified by the medical/scientific evidence. Instead of conducting this vote, it appears that the Review Panel, with your Vice President of Clinical Affairs present, voted on whether each recommendation warranted one of four actions: (a) no change was required, (b) no change was required with comment, (c) a revision was necessary or (d) a re-write was required. These voting parameters do not specifically appear anywhere in the AP, as even the secondary vote on what actions to take following the initial assessment of whether the evidence supported each recommendation does not permit a vote on whether to determine no change is warranted with the option to comment. Such an option actually diminishes the three permitted secondary voting options.

---

[1] See AP section IC3(a)
[2] Section IC3(a)
[3] Section IC4

My staff has notified the IDSA's Vice President of Clinical Affairs and your counsel of the improper voting procedure, and I understand it is the IDSA's position that the vote conducted was in accordance with the agreement and the AP. Moreover, the IDSA maintains this position even after my office sent a copy of a July 9, 2009 IDSA directive to the Review Panel's Chairperson and panel members instructing them as to the AP's voting requirements. This directive was drafted on IDSA letterhead and commanded adherence to the very two-stage voting process that the IDSA currently is at risk of violating. The directive (a copy of which I have enclosed for your consideration), stated that:

> "[e]ach Panel member must vote on each recommendation within the 2006 Guidelines" prior to a secondary vote in which "each Panel member must vote on an overall recommendation for the guidelines as follows: No changes are necessary, OR Sectional revision is needed; proposals for any such revisions should be made, OR Complete re-writing is needed."

The directive further restates the requirement that all votes "require supermajority support (75% or more), and specifies that "a minimum of seven panel members must vote in favor of a recommendation in order for the panel to deem it supported by the evidence, just as a minimum of seven panel members must support one of the three options for the overall guideline evaluation in order to recommend that option to the IDSA." [4] This directive highlights three important facts: (a) The IDSA actually agrees that the AP requires two separate voting processes and that the first involves an individual evaluation by each Review Panelist as to whether the scientific and medical evidence supports each recommendation in the 2006 guidelines; (b) that the Review Panel and its Chairperson were informed of this position prior to the scientific hearing and any voting activities, and thus should have followed the directive and (c) that the IDSA's Vice President of Clinical Affairs failed to admonish the Review Panel to follow the AP and directive during the voting phase and instead established a paradigm for that voting that violated the terms of the AP.

My staff has concluded from its review of the voting that the first recommendation, which states in part that "[d]iagnostic testing performed in laboratories with excellent quality-control procedures is required for confirmation of extra-coetaneous Lyme disease, HGA and babesiosis," received a vote to revise by four of the eight voting panelists on the Review Panel. The reasonable conclusion is that half of the eight panel members found that this provision was not supported by the medical/scientific evidence, such that it required revision and, consequently, that the requisite 75% supermajority could not be achieved in the primary votes to, in the IDSA's own words, "deem it supported by the evidence. . .."

---

[4] At the time the directive was written there were nine panelists, meaning a supermajority of seven panelists was required. Since that time, one panelist resigned from the panel for personal reasons, such that currently a supermajority would consist of six votes.

Since the Final Report has not yet been issued, a cure to this apparent violation in the form of an individual vote on whether each recommendation in the 2006 Lyme disease guidelines is medically/scientifically justified in light of all of the evidence and information provided" is readily achievable, especially since the Review Panel has already reviewed and considered the evidence presented. To date, the IDSA has rejected this relatively simple remedy.

I request that the IDSA hold an individual vote on whether each of the recommendations in the 2006 Lyme disease guidelines is medically/scientifically justified in light of all of the evidence and information provided, and to have that vote memorialized with particularity for the record, reported by the Review Panel Chairperson to the Ombudsman, and ultimately reflected in the final report as required by the AP.

Thank you for your attention to this most important matter.

Sincerely,

RICHARD BLUMENTHAL

RB/pas
